# Single‐Atom‐Enhanced Fully Inkjet‐Printed Electrochemical Sensor for Dopamine Detection

**DOI:** 10.1002/advs.76796

**Published:** 2026-07-27

**Authors:** Martin‐Alex Nalepa, David Panáček, Vítězslav Hrubý, Matěj Jendrišák, Rostislav Langer, Michal Langer, Petr Jakubec, Ivan Dědek, Vojtěch Kupka, Michal Mazur, Radek Zbořil, Michal Otyepka

**Affiliations:** ^1^ Regional Centre of Advanced Technologies and Materials Czech Advanced Technology and Research Institute (CATRIN) Palacký University Olomouc Olomouc Czech Republic; ^2^ Department of Physical and Macromolecular Chemistry Faculty of Science Charles University Prague Czech Republic; ^3^ Nanotechnology Centre Centre of Energy and Environmental Technologies VŠB – Technical University of Ostrava Ostrava‐Poruba Czech Republic; ^4^ Department of Materials Engineering and Recycling Faculty of Materials Science and Technology VŠB – Technical University of Ostrava Ostrava‐Poruba Czech Republic; ^5^ IT4Innovations VŠB – Technical University of Ostrava Ostrava‐Poruba Czech Republic

**Keywords:** dopamine, electrochemical sensor, inkjet printing, single‐atom catalyst, single‐atom sensing

## Abstract

Single‐atom (SA) engineering offers atomic‐level control over interfacial reactivity, yet the lack of printable SA‐based inks hinders its practical translation into electrochemical sensing. Here, a fully water‐based inkjet‐printable ink is introduced based on nitrogen‐doped graphene acid (NGA) hosting atomically dispersed Cu centers (NGA‐Cu‐ink). The ink enables digitally controlled, spatially defined deposition and fabrication of low‐cost ($0.04 per sensor), fully inkjet‐printed sustainable electrodes on paper. A comparison of NGA functionalized with different SA dopants (Cu, Mn, Fe, Ce) identifies a strong dopant‐dependent electrochemical response, with Cu uniquely enhancing the analyte signal while other dopants suppress it, demonstrating that SA identity is a decisive design parameter in printed sensing interfaces profiling sensitivity and selectivity. The functional role of the NGA support is to provide dense anchoring sites (nitrogen and carboxylate groups) that stabilize atomically dispersed metal centers and create adsorption‐ and electron‐transfer–active microenvironments. The NGA‐Cu‐ink yields enhanced dopamine oxidation, enabling quantitative detection on fully printed devices (limit of detection 9.7 µM; linear range 50–400 µM) and printed‐on‐electrode platforms (10.6 µM), while maintaining <10% signal variation over 11 weeks. The approach establishes a general route to single‐atom graphene‐based inks for scalable, reproducible, and low‐material‐consumption manufacturing of advanced electrochemical sensors.

## Graphical abstract

Fully inkjet‐printed paper‐based dopamine sensors are fabricated using nitrogen‐doped graphene acid functionalized with copper single atoms. The copper sites uniquely enhance dopamine oxidation through strong molecular adsorption, as verified by electrochemical measurements and DFT calculations. The approach highlights the potential of single‐atom inks for scalable, low‐cost manufacturing of high‐performance electrochemical sensors.

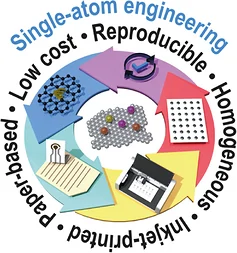

## 1 Introduction

Single‐atom‐based materials have emerged as a leading direction in nanomaterials research due to their unique combination of atomic dispersion, full metal atom utilization, and tunable electronic properties [1, 2]. In these systems, individual metal atoms are anchored onto solid supports, creating well‐defined coordination environments that differ fundamentally from those in bulk metals or nanoparticles [3, 4]. Single‐atom catalysts (SACs) have shown exceptional performance in electrocatalytic processes such as the oxygen reduction reaction (ORR) [5, 6], hydrogen evolution reaction (HER) [7, 8], and carbon dioxide reduction reaction (CO_2_RR) [9, 10]. Their activity and selectivity often exceed those of conventional materials, and because each atom participates in the reaction, efficiency is maximized [11]. These features have not only advanced the field of electrocatalysis but have also sparked interest in applying single‐atom (SA) design concepts to other areas, including energy storage [12, 13], biomedicine [14, 15], and chemical detection [16, 17].

Despite this progress, the integration of SACs into electrochemical sensing has received far less attention. This contrast is important because the properties that make SACs effective in electrocatalysis, i.e., controlled surface chemistry, sensitivity to the surrounding environment, and adjustable electronic structure, are equally valuable for sensor design [18, 19, 20]. A sensor interface based on SACs could, in principle, be engineered for (i) precise tuning of the local chemical environment, (ii) selective interactions with specific target molecules, and (iii) flexible combinations of different metal atoms and supporting 2D materials [21, 22, 23]. However, only a few examples of SAC‐based electrochemical sensors have been reported, and studies that directly compare different dopants or systematically explore sensor behavior are still rare [24, 25, 26, 27, 28]. The field remains open for deeper investigation, particularly in establishing how SA materials can be adapted for robust, selective, and reproducible sensing applications. Fundamental questions concerning the impact of SA doping on the structure–function relationships that govern electrochemical sensor performance remain to be answered.

In electrochemical sensors, these performance characteristics, namely, sensitivity and selectivity, are strongly dictated by the modification of the electrode surface with functional materials [29, 30, 31]. At the material level, performance is governed by the density, accessibility, and chemical nature of active sites, which together control both the intensity of the signal and the preference toward specific analytes [32, 33]. To date, these properties are most commonly tuned using nanostructured modifiers such as metal [34] and metal‐oxide [35] nanoparticles, doped carbons [36], and hybrid nanocomposites [37], where high surface area and catalytic or affinity sites are exploited to increase sensitivity and introduce a degree of selectivity. However, such systems typically comprise heterogeneous ensembles of active sites with broad distributions of coordination environments, making it difficult to relate a specific structural motif to the observed sensing behavior and to rationally optimize analyte‐specific interactions [38, 39]. SA materials represent a natural evolution of this concept: by anchoring isolated metal atoms in well‐defined coordination environments on a suitable support, they provide an opportunity to modulate both sensitivity and analyte‐dependent response with atomic precision [40, 41]. Realizing this potential in practical sensors, however, requires not only appropriate SA‐engineered materials but also electrode fabrication strategies that preserve atomic dispersion and ensure reproducible exposure of the active sites.

Traditional deposition techniques, such as drop‐casting or spray‐coating, often result in inhomogeneous coverage, material agglomeration, and poor adhesion, which can compromise the stability and reproducibility of measurements [42, 43]. Inkjet printing (IP) offers a reliable alternative, providing high‐resolution, digitally controlled deposition of materials with minimal waste [44, 45, 46]. By operating on a drop‐on‐demand principle, it enables precise placement of small volumes of ink, ensuring uniform film formation while reducing undesirable issues such as the coffee‐ring effect, surface fouling, and detachment of material during electrochemical measurements [47, 48, 49]. These characteristics are especially advantageous when working with advanced nanomaterials that require uniform spatial distribution. For SACs, in particular, it is essential to deposit the material in a way that preserves atomic dispersion and provides sufficient exposure of active sites for analyte‐specific interactions [50]. IP offers this level of control, allowing the fabrication of homogeneous sensing interfaces with well‐anchored, functional material layers [51]. While several studies have demonstrated the feasibility of nanomaterial‐based inks for inkjet‐printed sensors, including graphene derivatives [52, 53, 54, 55], MXenes [56, 57, 58], and transition metal dichalcogenides [59, 60, 61], the direct IP of SA‐doped materials has not yet been reported. Combining SA‐engineered materials with inkjet‐based patterning offers a new opportunity in sensor development, enabling the rational design of surfaces with tailored reactivity and reproducible fabrication in a scalable format.

Several studies have demonstrated that SACs can enhance the electrochemical detection of dopamine, a biologically relevant neurotransmitter with a well‐defined redox response [38, 62, 63, 64, 65]. These works typically attribute the improved performance to the presence of atomically dispersed metal centers, which provide a high density of accessible and reactive sites. In some cases, mechanistic explanations have been proposed, often relating to facilitated electron transfer, improved adsorption, or modulation of the local electronic structure [66, 67, 68, 69, 70]. However, most reported systems focus on a single type of metal dopant without clearly articulating the rationale behind its selection [71, 72, 73, 74, 75, 76, 77, 78]. Moreover, comparative studies that systematically evaluate different metal atoms within the same structural framework are notably scarce. This represents a significant gap, as the electronic structure, coordination geometry, and redox properties of different metal centers are expected to influence electrochemical performance in distinct ways [79]. Dopamine, with its stable and well‐characterized redox activity, serves as a suitable model analyte for such comparative studies, enabling consistent benchmarking of electron transfer behavior across different materials [80]. By addressing this underexplored aspect of SA sensing, a broader understanding of structure–function relationships can be established. Such insights are essential for moving beyond empirical material selection and toward the rational design of sensing platforms tailored to specific redox‐active targets [81, 82].

In this work, we compare the performance of atomically dispersed Cu, Fe, Mn, and Ce species anchored to nitrogen‐doped carboxylated graphene (NGA) [83] for electrochemical dopamine detection. All materials were synthesized using the same carbon scaffold, allowing for consistent evaluation of SA effects on electrochemical performance. For practical implementation, all single‐atom NGA (NGA‐SA) materials were formulated into inks compatible with IP technology, following the scheme shown in Figure 1a, and used for the fabrication of fully inkjet‐printed electrodes. To the best of our knowledge, this represents the first‐ever report of inkjet‐printable SA‐based ink. Among the tested NGA‐SA materials, NGA‐Cu demonstrated the most favorable characteristics, with both electrochemical experiments and density functional theory (DFT) calculations confirming copper as the most effective dopant for this particular sensing application. NGA‐Cu‐ink was further used to fabricate fully inkjet‐printed sensors, which demonstrated reliable performance in dopamine detection. By leveraging the main advantages of IP, namely high precision, rapid prototyping, and minimal ink consumption, we established a scalable process for producing a large volume of sensors. The resulting platform combines the chemical precision of SA materials with the manufacturing versatility of digital printing. Notably, the final production cost per printed electrode was calculated as $0.04, highlighting the potential for scalable and cost‐effective production of flexible sensors for point‐of‐care measurements (Figure 1b–d). Taken together, this work introduces a new class of SA‐doped inks for IP of functional, low‐cost, and fully inkjet‐printed electrodes, and showcases that atomic‐scale metal functionalization efficiently modulates sensitivity and selectivity of electrochemical sensors, opening new horizons for multiplexed sensing and advanced sensorics, including artificial tongue and nose.

**FIGURE 1:**
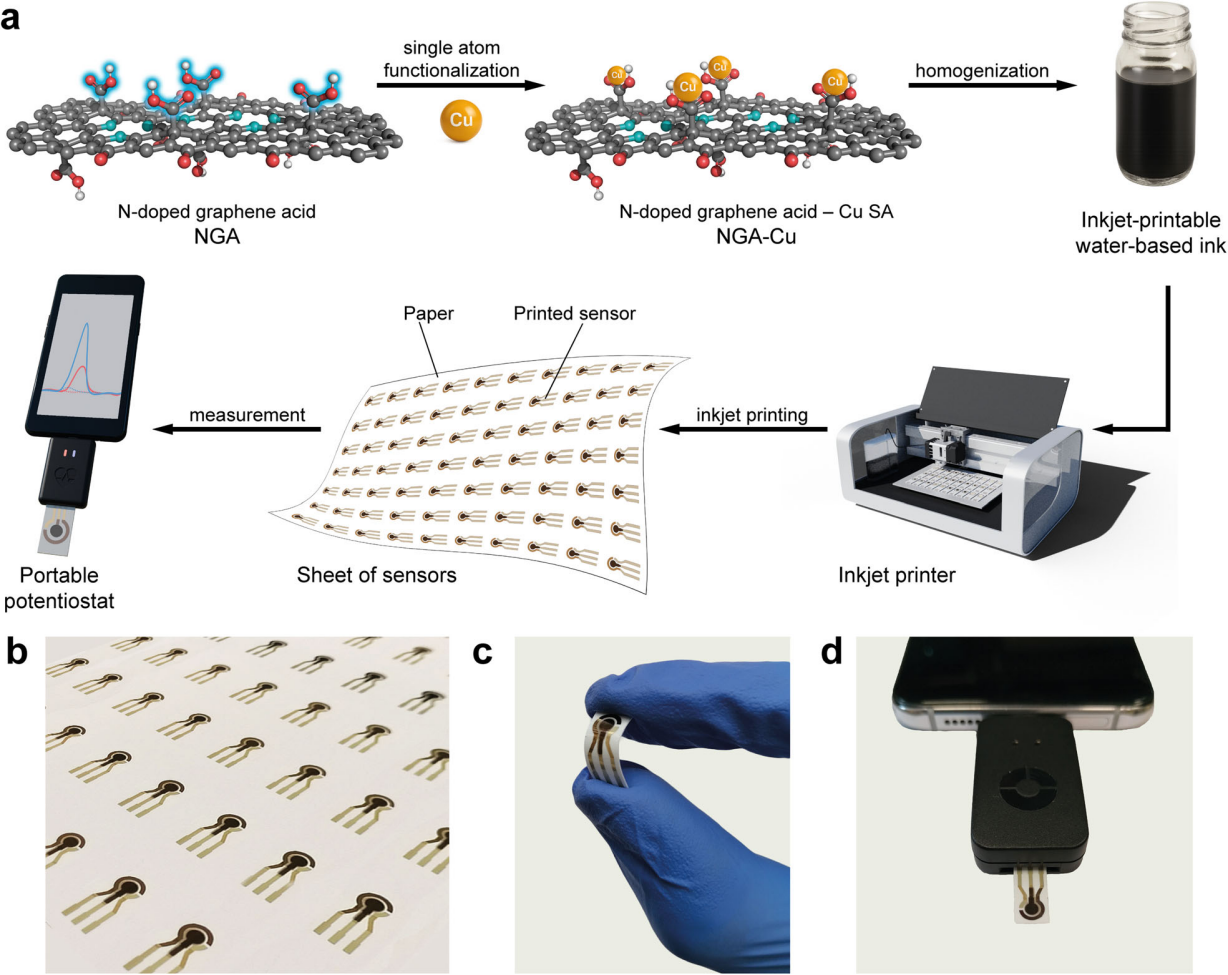
(a) Scheme of NGA‐SA‐ink synthesis and processing. NGA material is functionalized with a SA of choice and formulated into an inkjet‐printable ink. Such ink is then printed with the use of an inkjet printer onto a paper substrate to produce a large number of printed sensors, compatible with a portable potentiostat set‐up. Established printing process allows scalable (b) fabrication of flexible (c) sensors for point‐of‐care (d) measurements.

## 2 Results and Discussion

### 2.1 Chemical Characterization of NGA‐Ink and NGA‐Cu‐Ink

The NGA‐ink used in this study represents the sub‐450 nm fraction of NGA, obtained through a two‐step synthesis protocol described in previous work [83]. In brief, graphite fluoride was first sonicated in *N,N*‐dimethylformamide and it then reacted with sodium azide at 130°C. The resulting product was collected by filtration and thoroughly washed. The intermediate nitrogen‐doped graphene (GN3) material [84] was then subjected to oxidation in 45% aq. nitric acid to introduce abundant carboxylic groups. Following successive filtration and washing with deionized water, the oxidized product was dispersed in water, intensely sonicated, and purified via dialysis until a constant conductivity response. To isolate the colloidally stable fraction, the dispersion was further sonicated for 6 h and passed through a 450 nm membrane filter, yielding the final NGA‐ink (Figure S1). The rheological behavior of the resulting ink (Figure S2) was consistent with previously reported values [55]. NGA‐Cu‐ink was subsequently prepared by mixing the aqueous NGA‐ink dispersion with Cu(NO_3_)_2_ solution (as described in detail in the Materials and Methods, section 4.5). The same functionalization strategy was later applied to Mn, Fe, and Ce nitrate precursors to obtain the comparative NGA‐SA‐ink series.

Chemical characterization of both NGA‐ink and NGA‐Cu‐ink was performed to evaluate the material changes associated with the anchoring of atomically dispersed copper. Fourier transform infrared spectroscopy (FTIR) analysis of the solid content of NGA‐ink (Figure 2a) revealed vibrational features consistent with those reported in our previous studies [55, 83]. The characteristic fingerprint bands, typically associated with defective graphene networks as observed in other publications [84, 85], were obscured by more intense signals arising from oxygen‐containing moieties, indicating a high degree of functionalization. Specifically, the asymmetric stretching of carboxyl groups at 1605 cm^−1^ overlapped with otherwise visible aromatic C═C stretching near 1580 cm^−1^. Adjacent to this region, the carbonyl C═O stretching band at 1730 cm^−1^ appeared as the most intense feature. A distinct C─O stretching vibration was also observed at 1250 cm^−1^. At higher wavenumbers, a broad and intense band centered around 3400 cm^−1^ was assigned to O─H stretching vibrations, characteristic of strongly hydrogen‐bonded systems such as molecular carboxylic acids [55, 83]. This feature supports the notion that NGA‐ink exhibits properties similar to those of molecular carboxylic acids. Additionally, C─H stretching vibrations at 2930 cm^−1^, typically indicative of aliphatic hydrocarbons, were also apparent. Following copper coordination, the NGA‐Cu‐ink exhibited notable changes in its vibrational spectrum relative to the parent NGA material. Most significantly, the intensity of the C═O stretching band decreased, while two symmetric carboxylate‐related stretching bands in the 1340–1420 cm^−1^ region became more pronounced and shifted to lower wavenumbers. These changes indicate perturbation of the local carboxyl/carboxylate environment and are consistent with the involvement of oxygen‐containing groups, including carboxylate moieties, in Cu coordination.

**FIGURE 2:**
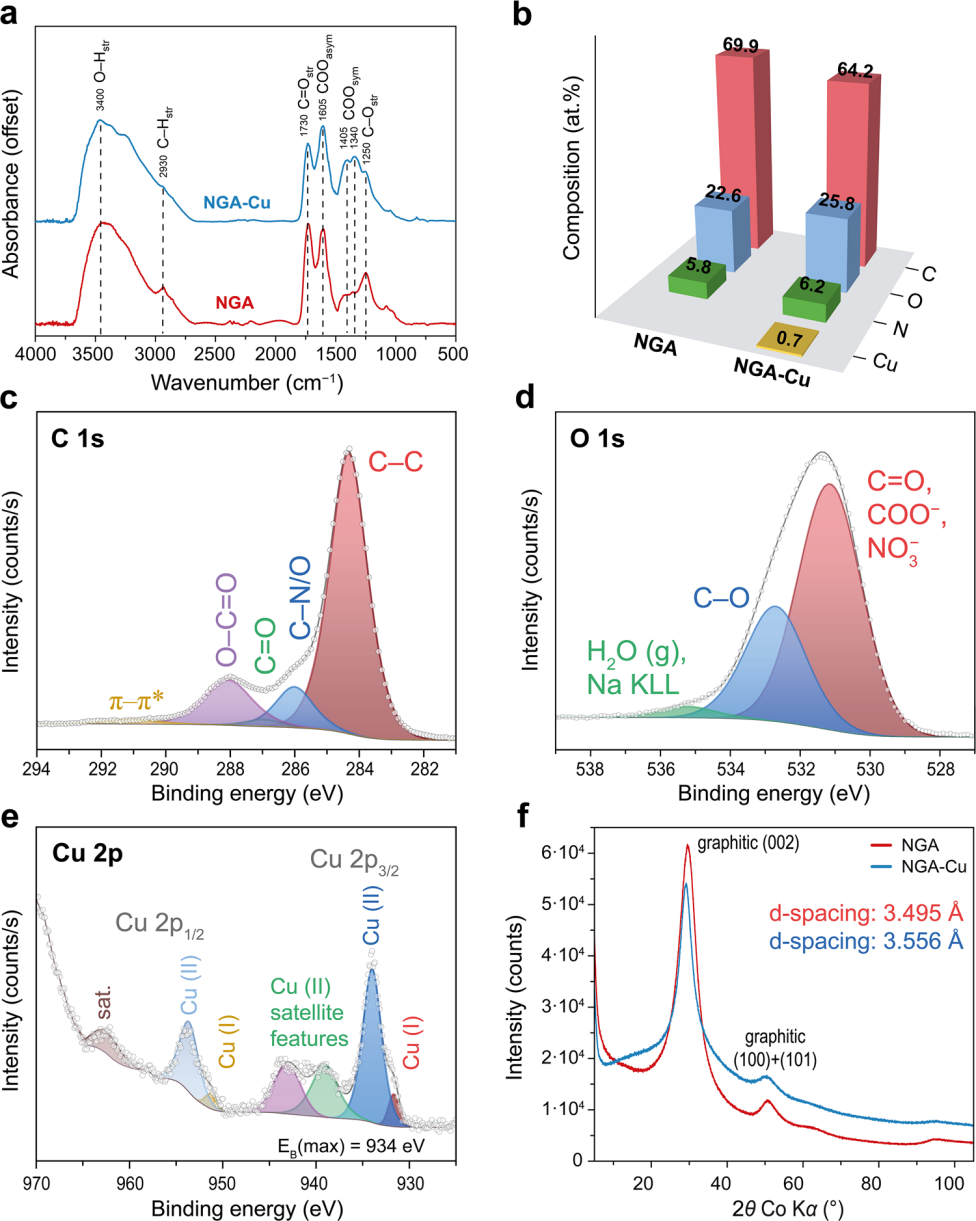
(a) FTIR spectra of NGA‐ink and NGA‐Cu‐ink with assigned vibration bands. (b) Atomic composition of NGA‐ink and NGA‐Cu‐ink. Deconvoluted HR‐XPS spectrum of (c) C 1s, (d) O 1s, and (e) Cu 2p of NGA‐Cu‐ink. (f) XRD spectra of NGA‐ink and NGA‐Cu‐ink with corresponding d‐spacing values.

X‐ray photoelectron spectroscopy (XPS) analysis of the solid NGA‐ink and NGA‐Cu‐ink (Figure 2b, see Figure S3a,b for XPS survey spectra and full atomic composition) showed that both materials were predominantly composed of carbon (69.9 and 64.2 at.%, respectively), oxygen (22.6 and 25.8 at.%, respectively) and nitrogen (5.8 and 6.2 at.%, respectively). The copper content in NGA‐Cu‐ink was 0.7 at.%, which correlates well with the 0.8 at.% determined by inductively coupled plasma mass spectrometry (ICP‐MS), confirming the successful incorporation of copper. The observed increase in oxygen and nitrogen content upon copper coordination suggests charge compensation by nitrate anions originating from the Cu(NO_3_)_2_ precursor. This interpretation was supported by the deconvoluted N 1s high‐resolution XPS (HR‐XPS) region (Figure S3c,d). The nitrate‐related component at 405.9 eV, already present in pristine NGA‐ink due to HNO_3_ treatment during NGA synthesis [55, 83], increased further after copper coordination. The rest of the N 1s envelope consisted of nitrogen species associated with acid‐treated nitrogen‐doped carbon under acidic conditions; specifically, graphitic nitrogen (∼401.3 eV) and a component at ∼399.4 eV representing predominantly pyrrolic nitrogen (399.8 eV), with a possible partial contribution from protonated pyridinic nitrogen (400.5 eV) [86]. In the C 1s regions (Figure 2c, Figure S3e), both HR‐XPS spectra were dominated by a peak between 284.3 and 284.9 eV, corresponding to C─C environments. Due to the complexity of the mixed hybridization system, this signal does not allow reliable distinction between sp^2^ and sp^3^ carbon [87, 88]. The second most intense peak, observed between 288.1 and 288.6 eV, corresponded to carboxyl groups [55, 83, 85]. Additional spectral components were assigned to other oxygenated carbon environments and C─N bonds, reflecting the nitrogen doping of the material. At higher binding energies, a minor π–π* shake‐up satellite was present, indicative of sp^2^ carbon domains. In the O 1s HR‐XPS spectrum, NGA‐ink (Figure S3f) exhibited comparable intensities for C═O and C─O components, in agreement with the presence of carboxylic functionalities. In contrast, for NGA‐Cu‐ink (Figure 2d), the low‐energy component at 531.1 eV became more pronounced, reflecting combined contributions from both C═O and nitrate groups, and possibly also from deprotonated carboxylate (COO^−^) species, which are known to contribute in this region [89]. The high‐energy peak at 535.2 eV was likely due to residual water vapor desorbing under ultra‐high vacuum conditions, or possibly from a sodium KLL Auger feature, given minor sodium contamination. The nature of the copper in NGA‐Cu‐ink was further probed via HR‐XPS. The Cu 2p region (Figure 2e) indicated a predominance of Cu^2+^ species, as evidenced by strong satellite features deconvoluted into components at 939.1 and 943.1 eV. The main Cu 2p_3/2_ peak at 934 eV was therefore assigned to Cu^2+^, while a shoulder at 931.7 eV was attributed to a minor Cu^+^ component. Analogous features were also present in the Cu 2p_1/2_ region. The Cu LMM Auger peak exhibited a kinetic energy maximum of 916.35 eV, derived from a binding energy maximum of 570.25 eV (Figure S4), measured using Al Kα radiation (hν = 1486.6 eV). The calculated modified Auger parameter (α′ = 1850.35 eV) is in excellent agreement with the literature value for Cu(NO_3_)_2_·3H_2_O (1850.49 ± 0.15 eV) [90]. Together, these findings confirm that copper is anchored in an ionic form, with its charge compensated by associated nitrate anions.

To further assess the state of the coordinated copper species, X‐ray diffraction (XRD) analysis was performed on the NGA‐Cu‐ink. As shown in Figure 2f, the diffraction pattern of NGA‐Cu‐ink closely resembled that of the pristine NGA‐ink, without any additional reflections attributable to crystalline copper or copper oxide phases. This absence of such reflections indicates that copper is not present as XRD‐detectable crystalline aggregates or nanoparticles, supporting its highly dispersed state within the NGA matrix. Notably, subtle differences were observed between the two patterns, with the most prominent being a slight broadening and shift of the graphitic (002) reflection. The interlayer spacing (d‐spacing), calculated from the diffraction maximum, increased from 3.495 Å (NGA‐ink) to 3.556 Å (NGA‐Cu‐ink). This expansion suggests that the coordinated Cu ions reside within or between the graphitic layers, likely interacting with the oxygen‐ and nitrogen‐containing functionalities. The broader profile of the reflection further implies increased local disorder [14, 91], consistent with the incorporation of isolated metal ions rather than ordered domains. Additional reduction experiments further showed that Cu‐related XRD reflections appeared only under strongly reducing conditions combined with elevated temperature, accompanied by TEM‐visible Cu‐rich aggregates (Figure S5a,b). These results support the strong retention of Cu species by the NGA framework in the as‐prepared NGA‐Cu‐ink.

Raman spectroscopy was employed to assess the structural disorder in both NGA‐ink and NGA‐Cu‐ink (Figure S6). The spectra of both materials exhibited characteristic D (∼1350 cm^−1^, associated with structural defects and disorder) and G (∼1580 cm^−1^, corresponding to in‐plane vibrations of sp^2^‐hybridized carbon) bands. In addition, both spectra showed a weak 2D band at ∼2675 cm^−1^ and a weak band at ∼2930 cm^−1^, assignable to the D+G combination mode [92]. The I_D_/I_G_ ratios of both materials were comparable, in the range of 1.6–1.7. These values reflect a high density of structural defects introduced during the nitric acid treatment used in NGA synthesis. Importantly, the negligible change in the I_D_/I_G_ ratio upon copper coordination indicates that the overall defect structure remains largely unaffected. The weak 2D band in both samples is consistent with the highly defective nature of the functionalized graphene framework and suggests that copper coordination does not substantially alter the overall graphitic structure.

### 2.2 Morphology of Printed NGA‐Cu‐Ink Structures

NGA‐ink is obtained by extensive sonication of NGA material followed by filtration through a 450 nm membrane. This processing step not only defines the final particle size distribution but also forms the dispersion suitable for both metal coordination and IP. To assess the flake morphology and confirm the atomic dispersion of copper, the solid content of NGA‐Cu‐ink was analyzed by high‐resolution transmission electron microscopy (HR‐TEM) and scanning transmission electron microscopy (STEM). Morphological analysis revealed that the solid phase consists of small, few‐layered graphene flakes generated through oxidative cutting of the GN3 precursor, as visible in Figure 3a. The inset displays a representative scanning electron microscopy (SEM) image of NGA‐Cu‐ink flake with a lateral dimension of approximately 350 nm, consistent with our earlier report on NGA‐ink [55] and confirming that copper coordination does not affect flake size or morphology. High‐angle annular dark‐field (HAADF) STEM imaging (Figure 3b,c) showed no evidence of copper clusters or nanoparticulate aggregates. Instead, sporadic bright contrast spots, consistent with atomic‐scale dimensions, were observed across the carbon matrix. These features were attributed to individual copper atoms coordinated to the NGA framework [93], as further supported by HAADF‐STEM contrast analysis (Figure S7). Elemental mapping by energy‐dispersive X‐ray spectroscopy (EDS) further confirmed the uniform composition of the material. The sample displayed a homogeneous spatial distribution of carbon atoms (Figure 3d) in the graphene network, which was closely followed by oxygen (Figure 3e) and nitrogen (Figure 3f) atoms (see Figure S8 for corresponding EDS spectrum). Importantly, copper atoms (Figure 3g,h) were evenly dispersed across the sample without any observable local aggregation.

**FIGURE 3:**
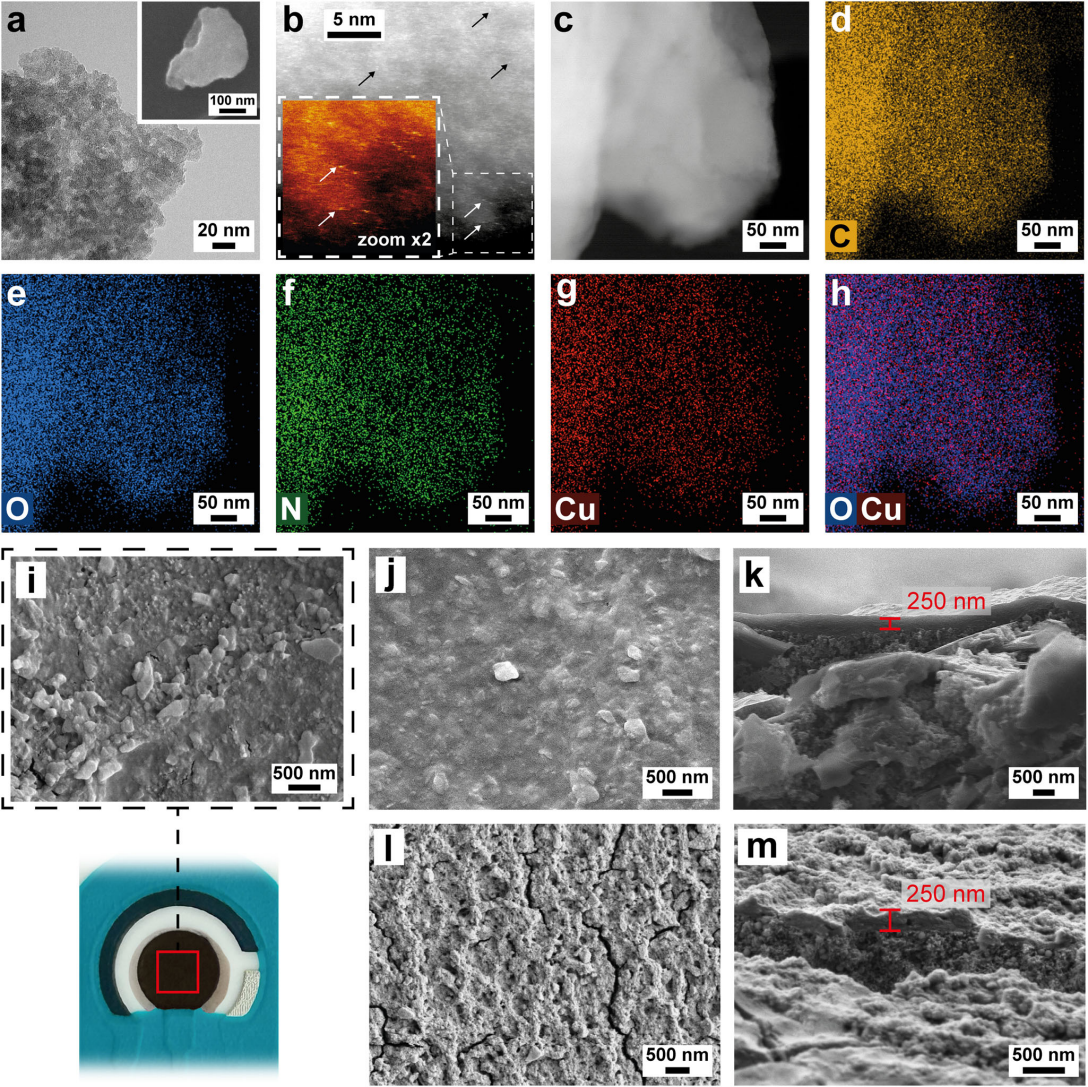
(a) TEM image of NGA‐Cu‐ink (inset: SEM image of NGA‐Cu‐ink). HAADF‐STEM images of NGA‐Cu‐ink: with visible atomically dispersed Cu centers (indicated by arrows) (b), as well as a general view (c) with corresponding EDS elemental mapping of (d) carbon, (e) oxygen, (f) nitrogen, (g) copper, and (h) combined oxygen and copper distribution in NGA‐Cu‐ink. (i) SEM image of five inkjet‐printed layers of NGA‐Cu‐ink on SPCE. SEM (j) and cross‐section SEM (k) images of twenty inkjet‐printed layers of NGA‐Cu‐ink on SPCE, with the printed film thickness marked in red. SEM (l) and cross‐section SEM (m) images of twenty inkjet‐printed layers of NGA‐Cu‐ink on photo paper, with the printed film thickness marked in red.

Once fully characterized, NGA‐Cu‐ink was loaded into a cartridge and inkjet‐printed via Fujifilm Dimatix DMP‐2850 printer onto two commercially available substrates to highlight the ink's versatility. First, the working electrode of a standard commercial screen‐printed carbon electrode (SPCE) was chosen, building on our previous findings that IP enables increased and more reproducible electrode response while reducing the required amount of deposited material compared to drop‐casting [49]. Second, a porous photo paper, PP‐201 (Canon), used for high‐resolution IP, was selected as a sustainable platform replacing ceramics used in SPCE. As shown in the SEM image in Figure 3i, five printed layers of NGA‐Cu‐ink were sufficient to densely and uniformly cover the porous SPCE surface across the entire working electrode area. Importantly, this approach achieved efficient material deposition using only ∼2.7 µg of material (corresponding to 1.35 µL of 2 mg·mL^−1^ NGA‐Cu‐ink), in contrast to ∼40 µg (20 µL) required to fully cover the electrode when applying the same ink by drop‐casting. Moreover, drop‐casting led to undesirable outcomes such as non‐uniform surface coverage and particle aggregation (Figure S9a,b), as well as detachment and release of graphene flakes into the electrolyte during electrochemical measurements (Figure S9c–e).

To further highlight the multilayer printing capability of SA‐based graphene inks, 20 layers of NGA‐Cu‐ink were printed onto the SPCE working electrode. SEM imaging revealed that while printing 10 (Figure S10a,b) or 20 layers (Figure 3j) no longer yielded individually resolvable flakes, the resulting film formed a dense and continuous coating. The topography of this coating followed the typical granular morphology of the bare SPCE surface (Figure S10c,d), including occasional larger features naturally present in the carbon paste. This morphology, although less defined, may be beneficial in applications where a compact graphene layer is desirable, such as in amplification layers in biosensors [94]. Cross‐sectional SEM analysis confirmed that the 20‐layer film reached a thickness of approximately 250 nm (Figure 3k).

Finally, to evaluate the ink's substrate versatility, the same 20‐layer printing procedure was applied to the commercial porous photo paper. The resulting SEM image (Figure 3l) again showed homogeneous surface coverage, although with slightly more textured morphology than on SPCE, reflecting differences in substrate structure and porosity. Nevertheless, the printed film reached a similar thickness of ∼250 nm (Figure 3m), demonstrating an excellent reproducibility of the printing process across distinct substrates. These results illustrate that NGA‐Cu‐ink, and more broadly, SA‐based graphene inks, can be reliably used to fabricate robust and scalable electrochemical and sensing platforms via a straightforward IP process.

### 2.3 Electrochemical Characterization of NGA‐SA Systems

Having established the atomic‐scale dispersion, morphology, and printability of NGA‐Cu‐ink as the representative SA‐based ink, the same NGA‐ink functionalization strategy was extended to Mn, Fe, and Ce to evaluate how the identity of the metal center influences interfacial charge‐transfer properties and overall electrochemical behavior. These four metals were chosen for their ability to bind to the nitrogen‐ and carboxyl‐functionalized surface of NGA‐ink in different oxidation states and for their distinct redox and catalytic behaviors [14, 95]. This selection spans chemically diverse regions of the periodic table and includes variations in oxidation state flexibility, atomic radii, and d‐/f‐electron configurations (Table S1).

Copper (Cu^+^/Cu^2+^) is a well‐known redox‐active transition metal that forms strong complexes with oxygen‐ and nitrogen‐based ligands, and has previously been associated with improved electron‐transfer kinetics in dopamine sensing systems [96]. Manganese (Mn^2+^), characterized by a stable high‐spin d^5^ configuration, is frequently employed in biomimetic redox catalysts and offers insight into the role of univalent dopants [14, 97]. Iron (Fe^2+^/Fe^3+^), as a prototypical multi‐valent redox mediator, represents both electrochemical versatility and relevance to biological redox systems [98]. Cerium (Ce^3+^), a redox‐flexible rare earth metal, stands apart in its capacity for oxygen vacancy formation and reversible Ce^3+^/Ce^4+^ cycling, which are features that influence interfacial electron transport through alternative catalytic pathways [99].

Five layers of NGA‐SA‐inks were inkjet‐printed onto SPCEs, as this number is sufficient for homogeneous surface coverage (Figure 3i) while having a low electrochemical background (Figure S11). This ensured uniform, reproducible coverage across all systems and provided a consistent platform for subsequent comparative electrochemical evaluation.

To gain detailed insight into the interfacial charge transfer properties of the modified electrode surfaces, electrochemical impedance spectroscopy (EIS) was employed using 5 mmol·L^−1^ K_3_[Fe(CN)_6_] in 0.1 mol·L^−1^ KCl as a redox probe. The resulting Nyquist plots (Figure 4a) exhibit well‐defined semicircular regions at high frequencies corresponding to charge transfer resistance (R_CT_), followed by linear Warburg diffusion tails in the low‐frequency region. The impedance data were fitted using a modified Randles equivalent circuit (inset, Figure 4a), comprising solution resistance (R_S_), constant phase element (CPE), R_CT_, and Warburg impedance (W) to model semi‐infinite diffusion. Triplicate EIS measurements on independently modified electrodes for each material (Figure 4b, Table S2) revealed an increase in R_CT_ across the tested materials. Starting with the bare SPCE, which exhibited the lowest R_CT_ (328 ± 41 Ω), the resistance increased upon modification with NGA‐ink (457 ± 7 Ω), likely due to partial disruption of π‐conjugation and surface functionalization. Among the metal‐functionalized systems, NGA‐Ce‐ink showed the lowest R_CT_ (548 ± 67 Ω), followed by NGA‐Fe‐ink and NGA‐Mn‐ink with similar R_CT_ response, and peaking with NGA‐Cu‐ink (985 ± 75 Ω), which presented the most significant resistance to electron transfer. The increase in R_CT_ is also evident in the Bode plots, particularly in the mid‐frequency range (Figure S12a). This trend may be rationalized by considering the Lewis acidity and coordination tendencies of the individual metal centers: more acidic and strongly coordinating ions like Cu^2+^ can form tighter interactions with carboxyl and nitrogen moieties, potentially reducing the number of accessible conductive pathways and increasing electronic barriers to the redox probe. The same trend was observed in the cyclic voltammograms recorded under identical conditions (Figure S12b), supporting the results obtained from the impedance analysis.

**FIGURE 4:**
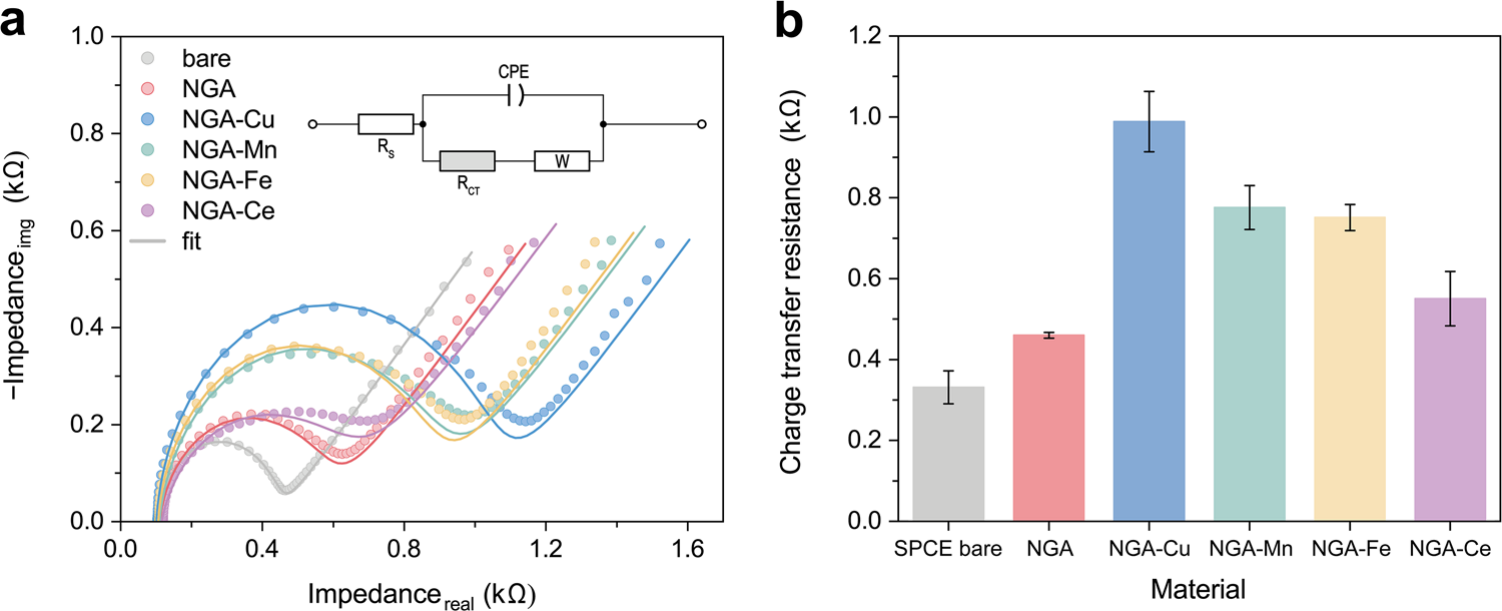
(a) Nyquist plots comparing EIS responses (5 mmol·L^−1^ K_3_[Fe(CN)_6_] in 0.1 mol·L^−1^ KCl) of bare SPCE and SPCEs modified with inkjet‐printed NGA‐ink variants (inset: modified Randles equivalent circuit used to fit the data), and (b) obtained R_CT_ values. The NGA‐SA materials in the column graphs are ordered by increasing SA oxidation state and the data are presented as mean ± SD from three independently prepared electrodes. Full electrochemical operating parameters are provided in Table S4.

To complement the impedance analysis, the heterogeneous electron transfer rate constants (k^0^) were determined from the obtained R_CT_ values. This approach provides a quantitative measure of the intrinsic electron transfer kinetics at the electrode–electrolyte interface and is particularly useful for evaluating the electrochemical activity of surface‐confined systems such as SACs [100]. Since R_CT_ is inversely proportional to the exchange current density (i_0_), according to equation [101]:

(1) $$R_{CT}=\frac{RT}{nFi_{0}}$$

where i_0_ is related to k^0^ via equation *i*_0_ =  *nFACk* ^0^ (where *n* stands for number of electrons transferred in the electrochemical reaction, *F* for Faraday constant, *A* for electrode area, *C* for redox probe concentration), it follows that

(2) $$R_{CT}=\frac{RT}{n^{2}F^{2}ACk^{0}}\;\;$$

The resulting k^0^ values are presented in Table S3. The bare SPCE exhibited the highest electron transfer rate (1.29·10^−3^ cm·s^−1^), which decreased upon surface modification with NGA‐ink (9.27·10^−4^ cm·s^−1^). Among the SA‐functionalized systems, NGA‐Ce‐ink maintained relatively high electron transfer activity (7.74·10^−4^ cm·s^−1^), while NGA‐Cu‐ink showed the lowest k^0^ (4.30·10^−4^ cm·s^−1^), indicating the strongest hindrance to ferricyanide redox kinetics.

Elimination voltammetry with linear scan (EVLS) [102] was employed to further dissect the total current responses of the NGA‐SA‐modified electrodes into their individual components, i.e., diffusion‐controlled, kinetic, and capacitive contributions. As shown in Figure S13, all materials exhibited dominant diffusion‐controlled signals, while the kinetic and capacitive components remained relatively minor. These results confirm that the voltammetric response of the ferricyanide redox system under the applied conditions is predominantly governed by mass transport. Nonetheless, the variation in R_CT_ and corresponding k^0^ observed across the SA‐functionalized electrodes reflects real differences in interfacial electron transfer kinetics.

### 2.4 Single‐Atom‐Enhanced Dopamine Detection

We further probed the effect of atomically dispersed metal centers on dopamine detection, since NGA itself can catalyze dopamine oxidation [55]. All four NGA‐SA‐ink variants were used to measure the dopamine response. Initial differential pulse voltammetry (DPV) measurements (dopamine 1 mmol·L^−1^ in phosphate‐buffered saline (PBS), pH = 7.4) were performed using electrodes modified by the conventional drop‐casting method (Figure S14). However, experiments with drop‐casted electrodes were hindered by multiple limitations, including inconsistent film formation, partial release of material into the electrolyte, and high capacitive background currents, which were particularly problematic in the low‐potential window where dopamine oxidation occurs. These issues resulted in poor signal resolution and unreliable quantification. It should be noted that the different nominal amount of deposited material also contributes to the comparison between drop‐casted and inkjet‐printed films. However, as recently shown [103] for the same NGA/SPCE system, the electrochemical response is not governed by deposited mass alone, but by how effectively the deposition method translates the active material into a continuous, wetted, and electronically coupled interface. By contrast, IP provided reproducible, uniform deposition while minimizing flake stacking. The DPV results obtained from inkjet‐printed NGA‐SA‐modified electrodes (Figure 5a, see Figure S15a–f for background signal in PBS) revealed a clear variance in their ability to catalyze dopamine oxidation. While all NGA‐based materials outperformed the unmodified SPCE, the NGA‐Cu‐ink exhibited a notable enhancement relative to the pristine NGA‐ink. Electrodes modified with Mn‐, Fe‐, or Ce‐functionalized variants consistently showed reduced peak currents. Apparent electrochemical surface area values (ECSA_app_, Table S5) were calculated from the cyclic voltammetry (CV) measurements in ferricyanide (Figure S12b) using the modified Randles–Ševčík approach for pseudo‐reversible systems [104] and are used only for comparative evaluation. The comparable ECSA_app_ values obtained for bare SPCE, NGA‐ink‐, and NGA‐SA‐ink‐modified electrodes indicate that the differences in dopamine response are not primarily governed by electroactive area, but rather by analyte‐specific interactions at the NGA‐SA interface.

**FIGURE 5:**
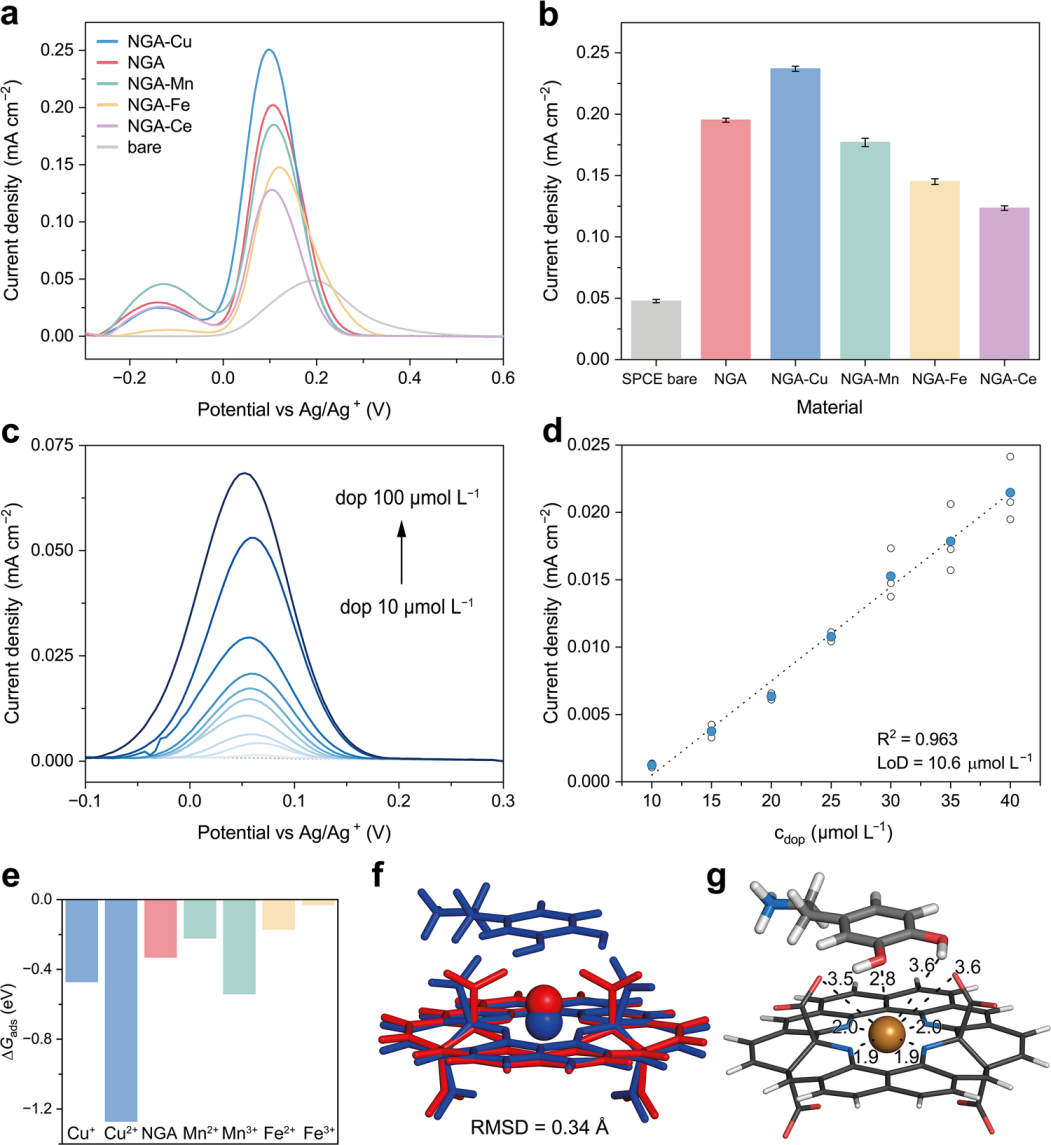
(a) DPV responses of bare SPCE and SPCEs modified with inkjet‐printed NGA‐ink variants in the presence of dopamine (1 mmol·L^−1^ in PBS buffer), and (b) corresponding peak current density values. The NGA‐SA materials in the column graphs are ordered by increasing SA oxidation state and the data are presented as mean ± SD from three independently prepared electrodes. (c) DPV responses of SPCEs modified with inkjet‐printed NGA‐Cu‐ink in the presence of PBS (blank) and dopamine at concentrations ranging from 10 to 100 µmol·L^−1^, and (d) calibration curve of the sensor; white points show individual values from triplicate measurements, while blue points show mean values for each dopamine concentration. Full electrochemical operating parameters are provided in Table S4. DPV responses were baseline‐corrected by polynomial fitting. (e) Adsorption free energies of dopamine at NGA‐SA in water. (f) RMSD analysis of NGA‐Cu^2+^ before (red) and after (blue) adsorption of dopamine. (g) Optimized structural model of adsorbed dopamine at NGA‐Cu^2+^ with bond lengths in Å.

The systematic decline in dopamine response (Figure 5b), from copper as the most active to cerium as the least, contrasts sharply with the trend observed in R_CT_ and k^0^ values, where NGA‐Cu showed the most hindered charge transfer in the ferricyanide system. Rather than correlating linearly with general electron‐transfer kinetics, the dopamine response appears to reflect a selective synergy between the redox‐active Cu centers and the oxygen/nitrogen‐functionalized conjugated graphene matrix. Such an interface likely facilitates both adsorption and oxidation of dopamine through favorable electronic coupling and catalytic activation. In this system, only copper provides the right balance of redox potential, coordination environment, and surface accessibility. In contrast, other atoms, despite their potential electroactivity, may perturb the local surface chemistry or active site architecture in ways that impair dopamine oxidation. Quantitatively, NGA‐Cu‐ink enhanced the dopamine signal by approximately +22% (0.236 mA·cm^−2^) compared to pristine NGA‐ink (0.194 mA·cm^−2^), while the most inhibiting system (NGA‐Ce‐ink) displayed a reduction of −37% (Table S6, all quantitative DPV comparisons were performed using three independently prepared electrodes for each material). This enhancement corresponds to NGA‐Cu‐ink containing approximately 4 wt.% Cu, as determined by XPS and ICP‐MS analysis. Additional DPV and CV measurements with lower Cu contents of 2.4 and 0.8 wt.% showed progressively weaker dopamine responses approaching that of pristine NGA‐ink (Figure S16), supporting the selected Cu loading as an effective compromise between electrochemical response and ink stability. Since NGA‐Cu‐ink was identified as the most effective dopant for dopamine oxidation, its pH‐dependent background response was further evaluated in blank Britton–Robinson buffer over the pH range 4–9 (Figure S17). The DPV and CV responses showed only gradual background variations across the pH range, without the emergence of distinct Cu‐related redox peaks or additional faradaic processes, indicating that the NGA‐Cu interface remains electrochemically stable under the tested pH conditions.

To further validate the sensing performance of NGA‐Cu‐ink for enhanced dopamine detection, DPV was carried out across a range of analyte concentrations. As shown in Figure 5c, the current density increased proportionally with dopamine concentration, yielding a well‐defined linear calibration curve (Figure 5d). This enabled the determination of key sensor performance parameters. The limit of detection (LoD) was calculated from the minimum detectable signal, defined as the mean blank response plus three times the sample standard deviation of the blank response:

(3) $$y_{LoD}={\bar{y}}_{blank}+3s_{b}$$

where *ȳ_blank_* is the mean current density of the blank response and *s_b_* is the sample standard deviation of the blank response. The corresponding dopamine concentration (*x_LoD_*) was then obtained from the calibration equation:

(4) $$y_{LoD}=a+b\cdot x_{LoD}$$

where *a* is the intercept and *b* is the slope of the calibration line. Using this approach, the LoD was determined to be 10.6 µmol·L^−1^. Sensitivity (S) was obtained by normalizing the slope (µA·µM^−1^) to the geometric surface area of the working electrode (cm^2^), yielding a value of 0.70 µA·µM^−1^·cm^−2^. Additionally, the sensor exhibited a linear dynamic range from 10 to 100 µmol·L^−1^, demonstrating its suitability for quantitative dopamine detection in this concentration window. The LoD and sensitivity values obtained are comparable to previously published works, which describe the use of SA materials based on N‐doped carbon for dopamine detection (Table S7).

To further substantiate the electrocatalytic behavior observed in DPV measurements, CV was performed in 1 mmol L^−1^ dopamine in PBS using bare SPCEs and SPCEs modified with inkjet‐printed NGA‐, NGA‐Cu‐, NGA‐Mn‐, NGA‐Fe‐, and NGA‐Ce‐inks (Figure S18). In contrast to the broad and weaker dopamine oxidation response of the bare SPCE, NGA‐Cu‐ink showed a well‐defined anodic peak shifted to less positive potential, together with the highest anodic current among the tested SA‐modified electrodes (Figure S18a). The presence of a corresponding cathodic response during the reverse scan and a smaller apparent peak‐to‐peak separation compared with the bare SPCE indicate improved reversibility of the dopamine/dopamine‐quinone redox couple at the NGA‐Cu‐ink interface [105, 106]. This behavior supports the conclusion that atomically dispersed Cu centers facilitate dopamine oxidation, rather than merely increasing the capacitive or background contribution. The weaker and less balanced responses of NGA‐Mn‐, NGA‐Fe‐, and NGA‐Ce‐inks further demonstrate that the enhancement is dopant‐specific, in agreement with the DPV data. The CV measurements also provide insight into possible secondary dopamine oxidation pathways. Under the applied sensing conditions, no additional peaks attributable to leucodopaminechrome/dopaminechrome, 5,6‐dihydroxyindole/indolequinone, or polydopamine‐type redox processes were observed [107]. Consecutive scans of NGA‐Cu showed stable, largely overlapping responses without progressive current decay (Figure S18b), indicating limited electrode passivation and no pronounced formation of polymeric dopamine‐derived layers within the timescale of the measurement [108]. Thus, dopamine oxidation on NGA‐Cu proceeds predominantly through the primary catechol/quinone redox process, while secondary cyclization or electropolymerization pathways are not pronounced within the investigated potential window. Together with the DPV comparison, these CV results identify NGA‐Cu as the most effective SA‐based ink for dopamine electrocatalysis and provide the experimental basis for the following DFT‐supported mechanistic analysis.

Electrochemical experiments showed that atomically dispersed Cu centers uniquely enhance dopamine oxidation at the NGA scaffold compared to other dopants. To understand this behavior mechanistically, DFT calculations were conducted. Dopamine adsorption was modeled on pristine NGA and metal‐anchored systems (Cu, Mn, Fe), providing atomistic insights into the interaction strength and coordination environment. Among all the examined systems, NGA‐Cu^2+^ exhibited the strongest affinity toward dopamine, with adsorption free energy (∆*G*_ads_) of −1.27 eV, followed by Cu^+^, Mn^3+^, pristine NGA, Mn^2+^, Fe^2+^, and Fe^3+^ (Figure 5e). The pronounced affinity of dopamine for NGA‐Cu, driven by π–π stacking with the NGA lattice and hydrogen bonding with COO^−^ groups of NGA, explains the experimentally recorded enhancement in dopamine sensing. In particular, the stronger adsorption of dopamine at the Cu‐modified surface increases its local concentration near electroactive sites, thereby enhancing the oxidation current observed experimentally.

A deeper look into the structural rearrangement of NGA‐Cu upon dopamine adsorption revealed notable structural changes, as evidenced by the root‐mean‐square deviation (RMSD) of 0.34 Å (Figure 5f). The average Cu─N bond length decreased from 2.2 to 1.9 Å, accompanied by an increase in the Cu binding energy within NGA from −5.02 to −5.36 eV, indicating stabilization of the Cu site upon dopamine adsorption. This rearrangement yielded a nearly planar Cu–N_4_ coordination environment (Figure 5g), in contrast to other NGA‐SA systems where SAs significantly protruded from the vacancy sites (Figure S19a–f). This finding was supported by Wiberg bond indices (WBI) analysis, which indicated an increase in Cu─N bond order in NGA‐Cu (from 0.2 to 0.3) upon dopamine adsorption. Interestingly, the nitrogen atoms help to stabilize the NGA itself, as their absence causes a distortion of the active site (Figure S19g,h).

Furthermore, carboxyl moieties near the Cu centers serve as critical anchoring sites that mediate dopamine adsorption on NGA‐Cu. Upon dopamine adsorption, the initial interaction between the Cu ion and nearby carboxyl groups (WBI = 0.3) is replaced by weaker hydrogen bonding between the hydroxyl groups of dopamine and carboxyl groups of NGA (WBI ≈ 0.1), significantly altering carboxyl's bonding characteristics. Concurrently, the O─H bond in dopamine also elongates from 0.9 to 1.0 Å, consistently with intramolecular hydrogen bonding and enhanced stabilization of the adsorbed molecule. In contrast, the adsorption of dopamine on graphene and N‐doped graphene with anchored Cu was considerably weaker, with ∆*G*_ads_ of −0.11 and −0.05 eV, respectively, highlighting the key contribution of COO^−^ in facilitating strong adsorption on NGA (Figure S19i,j). Taken together, these results identify NGA‐Cu as the most promising candidate among the studied SA‐based NGA systems for enhanced electrochemical dopamine detection, bridging experimental observations with mechanistic understanding of adsorption and catalytic activation.

### 2.5 Fully Inkjet‐Printed Paper‐Based Dopamine Sensor

With NGA‐Cu‐ink identified as the most promising candidate for enhanced dopamine detection among the tested SA‐functionalized materials, this system was subsequently employed in the fabrication of fully inkjet‐printed sensors. Using the DMP‐2850 printer, NGA‐Cu‐ink was deposited onto a photo paper substrate, alongside commercial metal‐nanoparticle‐based inks used for printing of conductive patterns (Figure 6a). The final design (Figure 6b) incorporated silver (Ag NPs) and gold (Au NPs) nanoparticle inks, along with a dielectric polymer ink, to produce miniaturized, paper‐based sensors with a total length of only 16 mm. The precise yet time‐efficient fabrication method minimized material waste and thus resulted in an exceptionally low final cost of $0.04 per electrode (including all ink and substrate consumption, see Table S8) while requiring only 1.3 µg (∼0.65 µL) of NGA‐Cu‐ink per sensor.

**FIGURE 6:**
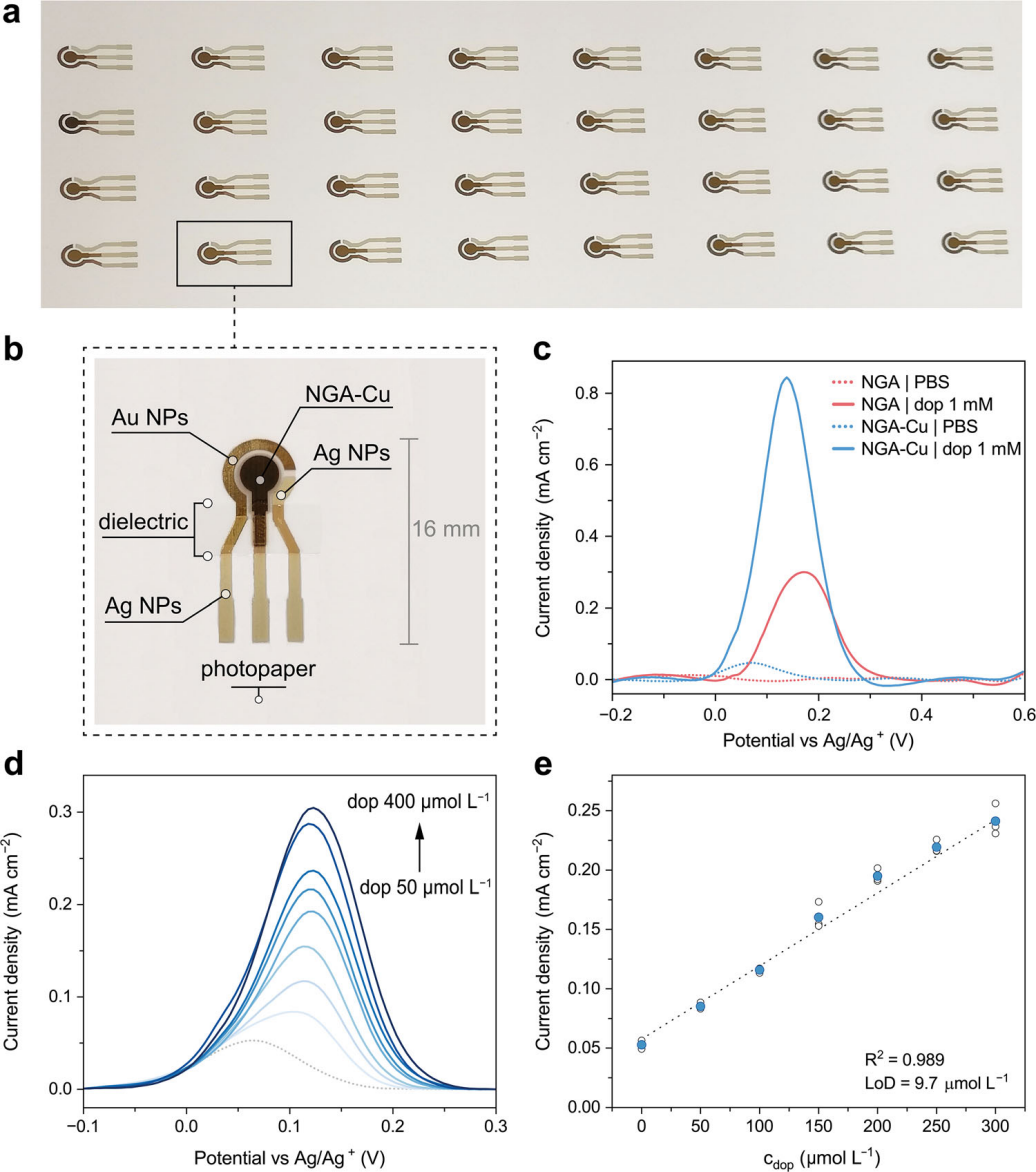
(a) Photo of a sheet of fully inkjet‐printed electrodes for dopamine sensing. (b) Photo of a single sensor consisting of Ag NPs (contacts and reference electrode), Au NPs (counter and working electrode), NGA‐Cu (working electrode) and a dielectric layer, inkjet‐printed onto photo paper. (c) Comparison of DPV responses of fully inkjet‐printed electrodes modified with NGA‐ink (red lines) and NGA‐Cu‐ink (blue lines) in the dopamine (1 mmol·L^−1^ in PBS buffer) and blank (PBS buffer) samples. (d) DPV responses of fully inkjet‐printed electrodes modified with inkjet‐printed NGA‐Cu‐ink in the presence of PBS (blank) and dopamine at concentrations ranging from 50 to 400 µmol·L^−1^, and (e) calibration curve of the printed sensor; white points show individual values from triplicate measurements, while blue points show mean values for each dopamine concentration. Full electrochemical operating parameters are provided in Table S4. DPV responses were baseline‐corrected by polynomial fitting.

To validate previous findings of the SA enhancement study in this fully inkjet‐printed configuration, DPV responses of sensors modified with NGA‐Cu‐ink and pristine NGA‐ink were compared under identical conditions. The same trend, the enhanced dopamine oxidation in case of the NGA‐Cu, was reproduced on the fully printed platform (Figure 6c), confirming the translatability of previously observed effects. Notably, the blank response of the fully inkjet‐printed NGA‐Cu sensor displayed a small oxidation feature at approximately +0.1 V, which was not clearly resolved in the SPCE‐based configuration. This feature is consistent with the electroactivity of Cu‐containing sites and is supported by additional CV measurements of the fully inkjet‐printed NGA and NGA‐Cu electrodes (Figure S20).

In addition, the response of fully inkjet‐printed sensors was evaluated toward selected electroactive interferents relevant to dopamine sensing, namely uric acid, serotonin, melatonin, and ascorbic acid [109]. For uric acid, serotonin, and melatonin, NGA‐Cu‐ink produced measurable oxidation signals; however, these responses were lower than those obtained with pristine NGA‐ink under identical conditions (Figure S21a–c). In the case of ascorbic acid, copper functionalization led to a modest increase in the oxidation response compared with NGA‐ink, although the signal remained substantially lower than that observed for dopamine (Figure S21d). Direct comparison of the individual NGA‐Cu‐ink responses further confirmed that dopamine produced the dominant signal among the tested molecules under the applied conditions (Figure S21e). These observations show that Cu incorporation does not act as a universal amplifier of all electroactive species, but rather modifies the analyte‐dependent response profile of NGA‐based electrodes, with a preferential enhancement toward dopamine. More broadly, these results suggest that the choice of SA dopant can be used to tune, at least partially, the relative response of NGA‐based electrodes toward different electroactive molecules.

DPV measurements across a range of dopamine concentrations yielded a proportional increase in current density (Figure 6d) and a well‐defined linear calibration curve (Figure 6e). Using the same equations, the value of LoD was determined to be 9.7 µmol·L^−1^. The sensitivity of the electrode was calculated as 0.62 µA·µM^−1^·cm^−2^. Moreover, a long‐term stability study conducted on a batch of identically printed sensors revealed sufficient reproducibility over the period of 11 weeks (Figure S22), with the dopamine response varying by less than 10% from the average (0.8 mA·cm^−2^).

## 3 Conclusion

In this work, we introduced a novel class of SA‐engineered graphene inks compatible with a marketed inkjet printer and demonstrated their value for the fabrication of fully inkjet‐printed paper‐based electrodes suitable for dopamine detection. A systematic electrochemical comparison of NGA‐ink functionalized with four different atomically dispersed metal centers (Cu, Fe, Mn, Ce) revealed that NGA‐Cu‐ink provides the most effective enhancement for dopamine detection. Specifically, NGA‐Cu‐ink achieved a +22% increase in current response compared to pristine NGA‐ink, while other dopants led to diminished performance. This result highlights the crucial role of dopant–analyte synergy in redox sensing, demonstrating that electron transfer resistance alone cannot predict sensing effectiveness. The NGA‐Cu‐ink formulation proved compatible with IP technology, enabling uniform, reproducible deposition with minimal material consumption and excellent printing precision. Building on this, we fabricated fully inkjet‐printed paper‐based sensors for enhanced dopamine detection. The total amount of NGA‐Cu‐ink used per electrode was only 1.3 µg, resulting in a low production cost of $0.04 per sensor. Long‐term stability testing confirmed signal retention within 10% variation over 11 weeks. These findings highlight the robustness of the IP process for scalable, low‐cost production of reliable electrochemical sensors, including paper‐based, flexible, and disposable formats, and demonstrate potential for utilizing SA material inks. Furthermore, the study shows that the selection of the SA shapes both the sensitivity and the analyte‐dependent response of NGA‐based electrodes, highlighting a key design principle in emerging SA sensing strategies. Overall, this approach establishes a practical and scalable route for integrating SA materials into next‐generation electrochemical sensors, designed with atomic precision to achieve enhanced sensing performance and open new horizons for affordable multiplex sensors.

## 4 Materials and Methods

### 4.1 Reagents and Materials

Graphite, fluorinated polymer > 61 wt.% F – GF, (Millipore Sigma), sodium azide 99% – NaN_3_ (Millipore Sigma), dimethylformamide pure – DMF (Lach:ner), absolute ethanol – EtOH (Penta), nitric acid 65% – HNO_3_ (Lach:ner) and acetone 99% (Millipore Sigma) were used for the synthesis. Aqueous stock solutions of copper [Cu^2+^], manganese [Mn^2+^], iron [Fe^3+^] and cerium [Ce^3+^] were prepared from the nitrate salt (p.a., Merck). Ultrapure water (18 MΩ·cm) was used for all solutions. Commercially available silver nanoparticle ink Sicrys I20DM‐206 (Ag content 20 wt.%, viscosity 5 mPa·s, surface tension 34 mN·m^−1^) was purchased from PV Nano Cell, Israel. Gold nanoparticle ink DryCure Au‐J 0410B (Au content 10 wt.%, viscosity 4 mPa·s) was acquired from C‐INK, Japan. Polymer dielectric ink InkOrmo‐7mPas (viscosity 7 ± 1 mPa·s) was purchased from micro resist technology, Germany. Photo paper PP‐201 (thickness 270 µm) was acquired from Canon, Czech Republic.

### 4.2 Synthesis of Nitrogen‐Doped Graphene (GN3)

The synthesis of GN3 was performed according to the previous report [84]. 10 g of fluorographite was dispersed in a glass flask in 300 mL of DMF and then stirred for 72 h, and sonicated for 4 h. 30 g of NaN_3_ was then added to the previous mixture, transferred to spherical glass flask and stirred and heated at 130°C for 3 days with a condenser. After cooling down, the solid product was washed with DMF (3x), acetone (3x), ethanol (3x), distilled water (3x) and hot distilled water (2x), using centrifugation (14000 rcf).

### 4.3 Synthesis of Nitrogen‐Doped Carboxylated Graphene (NGA)

The synthesis of NGA was performed according to the previous report [83], but using 45% of HNO_3_ instead of 65%. An amount of the previously prepared GN3 derivative was treated with 45% HNO_3_ for 48 h at 130°C in a glass flask with the condenser. After completion of the reaction, the product was purified by washing with filtration (Whatman cellulose membrane filter, pore size 0.2 µm); hot distilled water (5x), distilled water (5x) and purified by dialysis (dialysis tubing cellulose membrane, 14 kDa cutoff).

### 4.4 Synthesis of Nitrogen‐Doped Carboxylated Graphene Ink (NGA‐Ink)

NGA‐ink is a <450 nm fraction of NGA material. It was prepared as follows: freshly synthesized NGA material was dispersed in water and subjected to sonication for 6 h and then filtered through a 450 nm filter. The final concentration of solid component in the NGA‐ink was 2.0 mg·mL^−1^. Other ink‐related physical parameters such as surface tension and viscosity corresponded well to the previously published work [55].

### 4.5 Synthesis of Single‐Atom‐Functionalized NGA‐ink (NGA‐SA‐ink)

To functionalize NGA‐ink with different metal centers, a 2 mL aqueous suspension containing 4 mg of NGA was mixed with 16 µL of aqueous solutions of respective nitrate salts (0.1573 mol·L^−1^ Cu(NO_3_)_2_, 0.1816 mol·L^−1^ Mn(NO_3_)_2_, 0.1795 mol·L^−1^ Fe(NO_3_)_3_, 0.0714 mol·L^−1^ Ce(NO_3_)_3_), resulting in loading of 4 wt.% with respect to solid ink content. Each mixture was then rotated for 48 h at room temperature in the dark.

### 4.6 Characterization Methods

XPS measurements were performed using a Nexsa G2 spectrometer (Thermo Fisher Scientific, USA), equipped with an Al K*α* radiation source. The resulting data were analyzed with Avantage software.

FTIR spectra were obtained with an iS5 FTIR spectrometer (Thermo Nicolet, USA), which included a Smart Orbit ATR accessory featuring a ZnSe crystal. For the measurement, 3 µL of the sample dispersed in ultrapure water were applied to the ZnSe crystal and allowed to air‐dry.

XRD was measured using Aeris diffractometer (Malvern PANalytical, United Kingdom) in Bragg–Brentano parafocussing geometry. The diffractometer is equipped with an iron‐filtered Co K*α* radiation source and PixCell detector. The powder material was equally spread on a zero‐background Si slide and slightly pressed with glass to reach the required position in the sample holder. The XRD patterns were captured in 2*θ* range from 5° to 105° with total counting time 64 min for each pattern.

Raman spectra were collected using a Raman microscope (Thermo Fisher Scientific, USA) equipped with a laser 785 nm. The laser power on the sample was set to 2 mW with an exposure time of 1 s and 100 exposures per spectrum. Spectra were measured at 5 individual positions, and the averaged spectrum with corrected fluorescence is presented.

HR‐TEM images were conducted by HR‐TEM TITAN 60–300 microscope with an X‐FEG type emission gun, operating at 300 kV. STEM‐HAADF was performed with a HR‐TEM TITAN microscope operating at 80 kV. STEM combined with EDS was used for elemental mapping. These measurements were performed using a JEOL JEM NEOARM‐200F microscope equipped with a Schottky‐type field emission gun operating at 200 keV. JEOL JED‐2300 EDS spectrometer was used for elemental analysis. SEM images were obtained by the Jeol‐7900F SEM microscope, with an accelerating voltage of 5 kV.

### 4.7 Electrochemical Measurements

All electrochemical experiments were conducted using a Metrohm Autolab PGSTAT128N potentiostat (MetrohmAutolab B.V., Netherlands) controlled by the NOVA software package (version 2.1) and PalmSens Sensit Smart (PalmSens, Netherlands) controlled by the PStouch software (version 2.8). The experimental setup employed a three‐electrode configuration, utilizing either commercially available Metrohm DropSens SPCE or our inkjet‐printed electrode. All electrochemical experiments were carried out at room temperature (22 ± 2°C) in 0.01 mol·L^−1^ PBS (pH = 7.4), unless stated otherwise. The detailed operating parameters for each electrochemical measurement, including DC potential, frequency range, AC amplitude (EIS), scan rate, step potential, number of scans (CV), modulation amplitude, step potential, modulation time (DPV) and other relevant parameters are provided in Table S4.

### 4.8 Modification of SPCEs

#### 4.8.1 Modification via Drop‐Casting

Modification process of SPCEs via drop‐casting was performed as follows: 20 µL droplet of a powder suspension (2 mg·mL^−1^) was pipetted onto the working electrode surface, and it was subsequently allowed to dry at room temperature, forming a thin film.

#### 4.8.2 Modification via IP

Modification of SPCEs via IP technology was conducted using Fujifilm Dimatix Materials Printer, model DMP‐2850 equipped with Dimatix Drop Manager v3.2.4.2 software. Samba cartridges featuring piezoelectric printheads with 12 jets and a native drop volume of 2.4 pL were used. All layers were printed in 2540 dpi resolution, which corresponds to 1.7° head angle and drop spacing of 10 µm. The cartridge temperature was left at the default setting of 28°C. The cartridge print height was set to be 1 mm. Before printing the NGA(‐SA)‐ink layer, the jetting waveform and the voltage applied to the jets were optimized to form spherical drops without ligaments or satellite droplets during jetting. Only one jet at a time was used to ensure maximum printing quality. After each layer was printed, there was a 10 min pause to allow the printed ink to dry completely. For electrochemical characterization of NGA‐SA materials, five layers of corresponding inks were inkjet‐printed onto SPCE.

### 4.9 Inkjet Printing of Fully Inkjet‐Printed Electrodes

The printing process was performed using Fujifilm Dimatix Materials Printer, model DMP‐2850 equipped with Dimatix Drop Manager v3.2.4.2 software. Samba cartridges featuring piezoelectric printheads with 12 jets and a drop volume of 2.4 pL were used. All patterns were printed in high 2540 dpi resolution, which corresponds to 1.7° head angle and drop spacing of 10 µm. The cartridge temperature was left at the default setting of 28°C and the platen heating was turned off, keeping the substrate temperature at ambient level. The cartridge print height was set to be 1 mm. Silver nanoparticle ink was shaken for 2 h and filtered with a 1 µm nylon filter before filling the cartridge. Gold nanoparticle ink was shaken for the same amount of time and filtered with a 1 µm nylon filter. Photo paper dedicated for high‐resolution IP was used as a substrate.

Before printing each ink, the jetting waveform and the voltage applied to the jets were optimized to form spherical drops without ligaments or satellite droplets during jetting. Only one jet at a time was used to ensure maximum printing quality. First, the contacts, which are used to connect each electrode with the measuring device through a conductive channel, were printed together with a reference electrode using silver nanoparticle ink. Subsequently, the counter and working electrode were printed using gold nanoparticle ink. There was a 2 mm overlap between silver and gold patterns to ensure a conductive connection between them. After the printing of the gold patterns was finished, the printed parts were allowed to dry completely for half an hour before continuing with the process. In the next step, each printed electrode was flashed once with an external camera flash from a height of 1 cm above the surface. This step was performed to achieve photonic curing of printed metal patterns, causing the sintering of the silver and gold nanoparticles and increasing the conductivity of the printed patterns. Afterward, NGA(‐Cu)‐ink was printed onto the working electrode. To ensure good coverage of the gold surface, 3 layers of NGA(‐Cu)‐ink were printed over each other. After each layer was printed, there was a 10‐min pause to allow the printed ink to dry completely. The dielectric strip of 3.5 mm width, consisting of 3 layers of polymer dielectric ink, was printed last. The complete electrodes were then placed and stored in a vacuum desiccator until the measurement to be sure that all their components are perfectly dry. Prior to electrochemical measurements, the electrodes were cut out in such a shape that they fit into the appropriate adapter.

### 4.10 Computational Details

A finite‐size NGA model containing a double vacancy coordinated with four nitrogen atoms was used, with SAs embedded in the center of the vacancy (NGA‐SA) (Figure 5g and Figure S19). To account for different oxidation states of SAs, Cu^+^, Cu^2+^, Mn^2+^, Mn^3+^, Fe^2+^, and Fe^3+^ were modeled at their most stable spin multiplicities (Figure S19). As the electrochemical experiments were performed at pH 7.4, the deprotonated NGA scaffold was modeled together with a protonated NH_3_^+^ group of dopamine.

All investigated structures were optimized within the framework of DFT using the unrestricted *ω*B97X‐D functional [110] in combination with the Karlsruhe basis set def2‐SVP [111], as implemented in Gaussian 16 [112]. Solvent effects were included through the universal solvation model based on electron density (SMD) with a relative permittivity of 79, to mimic the PBS environment used in the electrochemical measurements [113]. Gibbs free energies were evaluated at 298 K and 1 atm employing the rigid‐rotor, harmonic‐oscillator, and ideal‐gas approximations within the framework of statistical thermodynamics. The binding energies Δ*G_b_* of SA at NGA were evaluated as: Δ*G_b_* = *G*_*NGA* − *SA*_  − *G_NGA_* − *G_SA_*, where *G*_*NGA* − *SA*_, *G_NGA_*, and *G_SA_* represent the thermal free energies of NGA with embedded SA, freestanding NGA, and isolated SA (SA = Cu, Mn, Fe), respectively.

The adsorption of dopamine to NGA was calculated using the triple‐zeta def2‐TZVP basis set. Due to the higher computational cost of frequency calculations at the triple‐zeta level, with respect to the used models, adsorption free energies were estimated as: $\Delta G=E_{SCF}^{def2-TZVP}+G_{thermal}^{def2-SVP}$, where $E_{SCF}^{def2-TZVP}$ denotes electronic energies at the def2‐TZVP level, and $G_{thermal}^{def2-SVP}$ represents thermal corrections derived from vibrational frequency calculations at the def2‐SVP level. The adsorption free energies of dopamine at NGA‐SA were evaluated as: Δ*G_ads_* = *G*_*dopamine*@*NGA* − *SA*_  − *G*_*NGA* − *SA*_ − *G_dopamine_*, where *G*_*dopamine*@*NGA* − *SA*_, *G*_*NGA* − *SA*_, and *G_dopamine_* represent the thermal free energies of the dopamine adsorbed at NGA‐SA, freestanding NGA‐SA, and isolated dopamine, respectively.

WBI were employed to determine bond orders before and after dopamine adsorption [114, 115].

## Author Contributions

**M.‐A.N**.: conceptualization; methodology; investigation; formal analysis; visualization; writing – original draft; writing – review & editing; **D.P**.: conceptualization; supervision; methodology; visualization; writing – review & editing; **V.H**.: investigation; visualization; writing – original draft; writing – review & editing; **M.J**.: investigation; **R.L**.: formal analysis; methodology; visualization; writing – original draft; writing – review & editing; **M.L**.: formal analysis; methodology; visualization; writing – original draft; writing – review & editing; **P.J**.: writing – review & editing; **I.D**.: writing – review & editing; **V.K**.: investigation; writing – review & editing; **M.M**.: investigation; visualization; **R.Z**.: writing – review & editing; **M.O**.: conceptualization; supervision; funding acquisition; writing – review & editing.

## Conflicts of Interest

M.O. has a share in biosimulation company InSiliBio (France) and supercaps company ATOMIVER (Czechia).

## Supporting information




**Supporting File**: advs76796‐sup‐0001‐SuppMat.docx.

## Data Availability

The data supporting the findings of this study are available in Zenodo at https://doi.org/10.5281/zenodo.21314113 (reference number 21314113).
