# Supplementary material for: Single‐Atom‐Enhanced Fully Inkjet‐Printed Electrochemical Sensor for Dopamine Detection
Source: Adv Sci (Weinh). 2026 Jul 27:e76796. Online ahead of print. doi: 10.1002/advs.76796 (PMC13403738; doi:10.1002/advs.76796)
Supplement: Supplementary file 1 — Supporting File: advs76796‐sup‐0001‐SuppMat.docx. [file ADVS-9999-e76796-s001.docx]

Supporting Information

To

Single-atom-enhanced fully inkjet-printed electrochemical sensor for dopamine detection

*Martin-Alex Nalepa^a,b^, David Panáček^a,c,*^, Vítězslav Hrubý^a^, Matěj Jendrišák^a,d^, Rostislav Langer^e^, Michal Langer^e^, Petr Jakubec^a^, Ivan Dědek^a^, Vojtěch Kupka^a^, Michal Mazur^b^, Radek Zbořil^a,c^, Michal Otyepka^a,e,*^*

^a^Regional Centre of Advanced Technologies and Materials, Czech Advanced Technology and Research Institute (CATRIN), Palacký University Olomouc, Šlechtitelů 241/27, 78371 Olomouc, Czech Republic

^b^Department of Physical and Macromolecular Chemistry, Faculty of Science, Charles University, Hlavova 2030/8, 12843 Prague, Czech Republic

^c^Nanotechnology Centre, Centre of Energy and Environmental Technologies, VŠB – Technical University of Ostrava, 17. listopadu 2172/15, 70800 Ostrava-Poruba, Czech Republic

^d^Department of Materials Engineering and Recycling, Faculty of Materials Science and Technology, VŠB – Technical University of Ostrava, 17. listopadu 2172/15, 70800 Ostrava-Poruba, Czech Republic

^e^IT4Innovations, VŠB – Technical University of Ostrava, 17. listopadu 2172/15, 70800 Ostrava-Poruba, Czech Republic


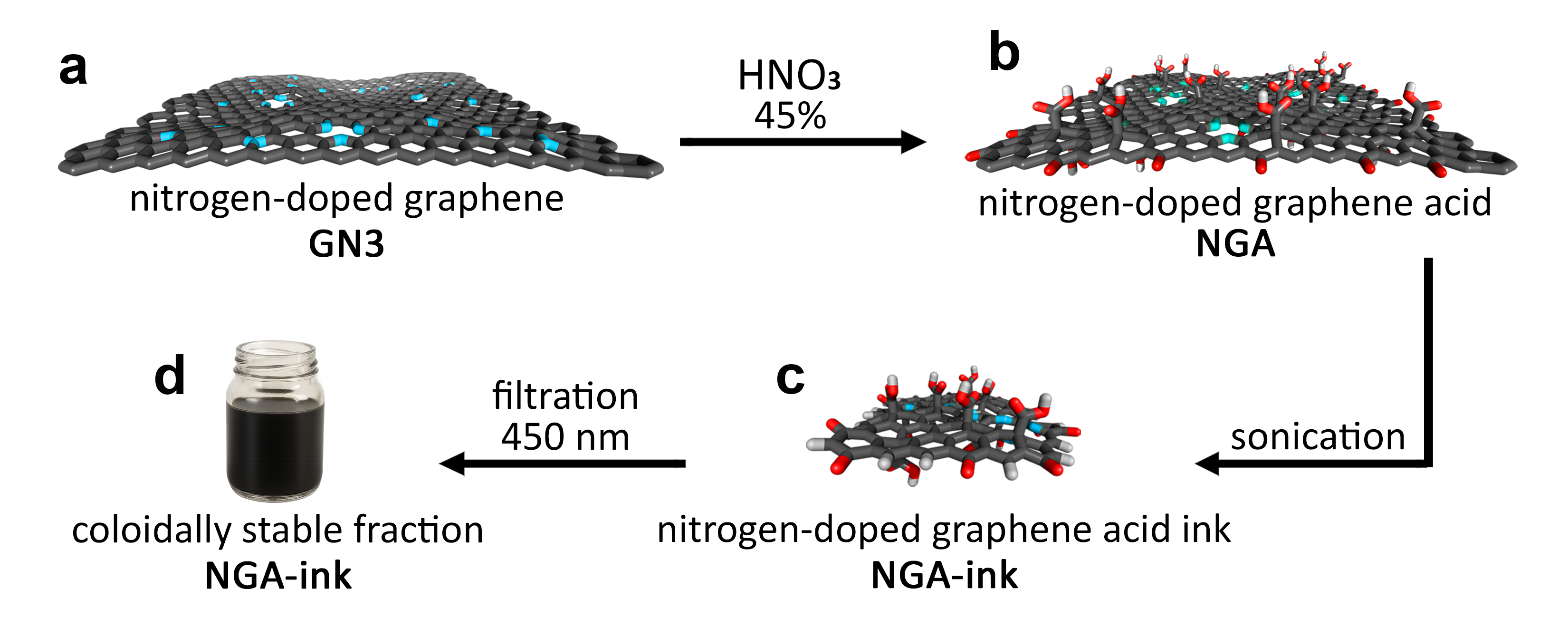


**Figure S1.** Synthesis of NGA-ink. Nitrogen-doped graphene (a) is subjected to oxidation in 45% nitric acid to obtain nitrogen-doped graphene acid (b). Following filtration, washing, sonication, and dialysis steps, the dispersion is further sonicated for 6 hours, yielding NGA-ink dispersion (c), which is then passed through a 450 nm membrane filter to obtain a colloidally stable fraction (d) used for inkjet printing.


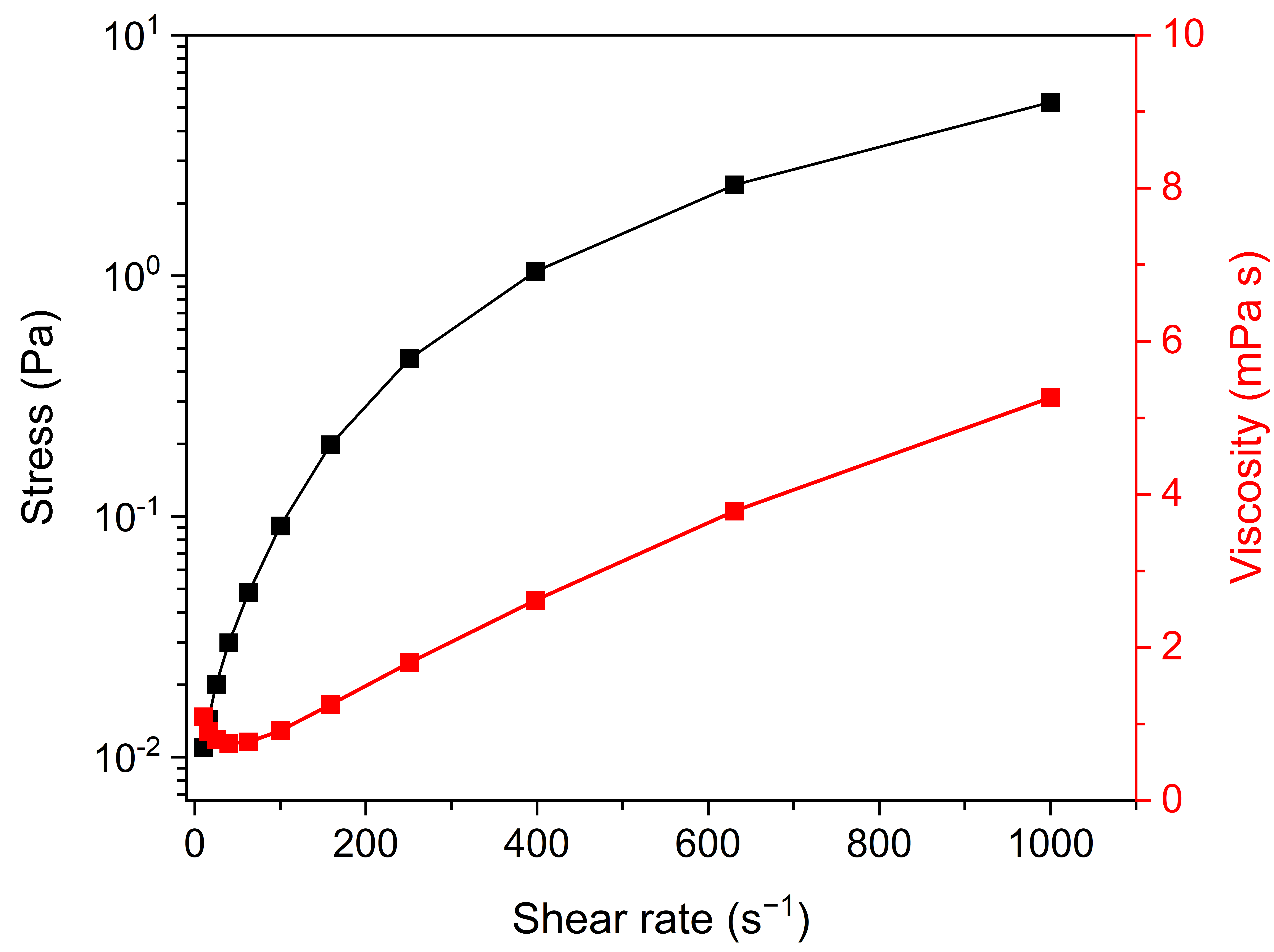


**Figure S2.** Rheological properties of the NGA-ink obtained by flow curve measurement.





**Figure S3.** Survey spectra (a) and full atomic composition (b) of NGA-ink and NGA-Cu-ink. Deconvoluted HR-XPS spectrum of c) N1s (NGA-ink), d) N1s (NGA-Cu-ink), e) C1s (NGA-ink), and f) O1s (NGA-ink).


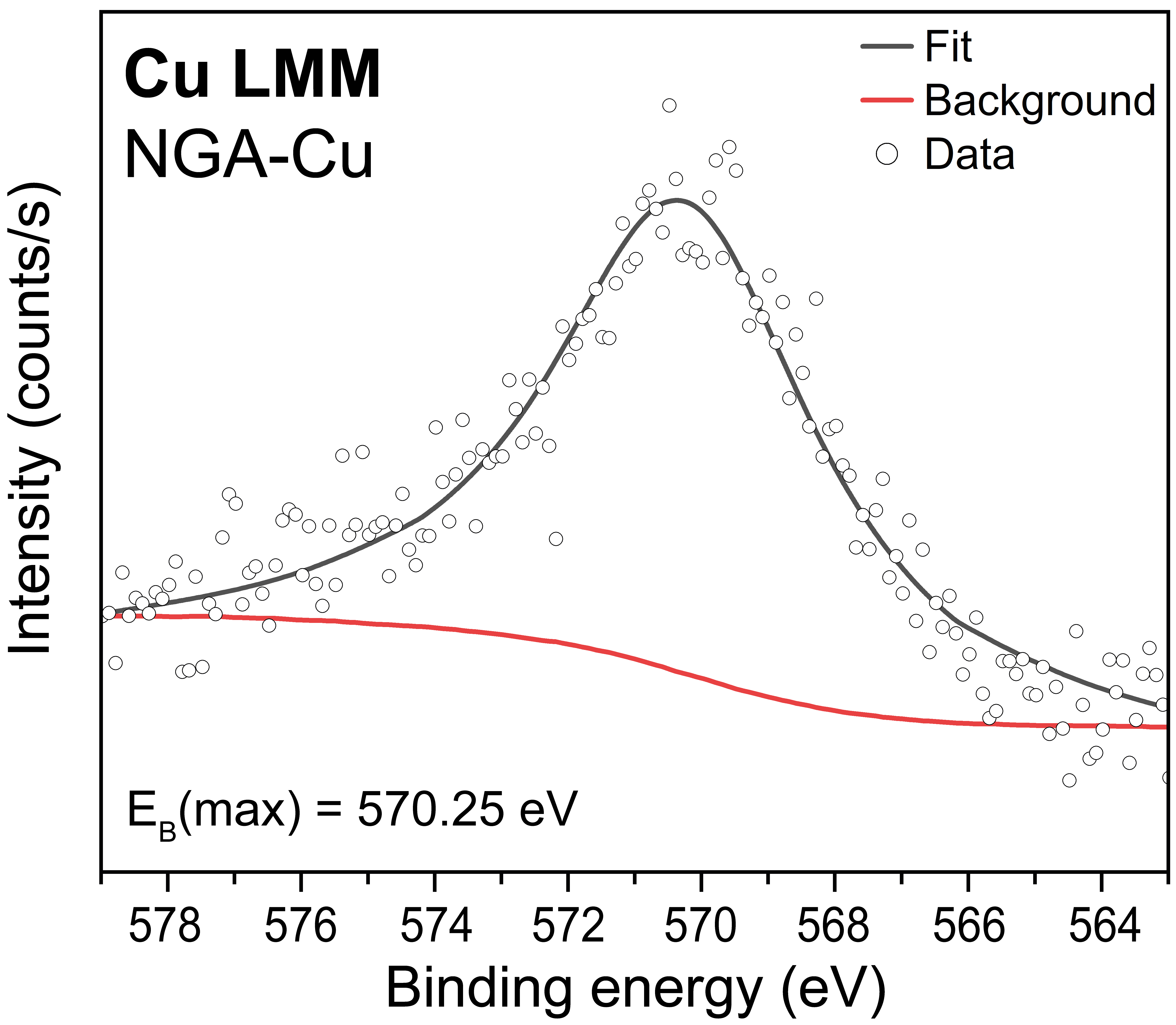


**Figure S4.** HR-XPS spectrum of Cu LMM Auger peak (NGA-Cu-ink).


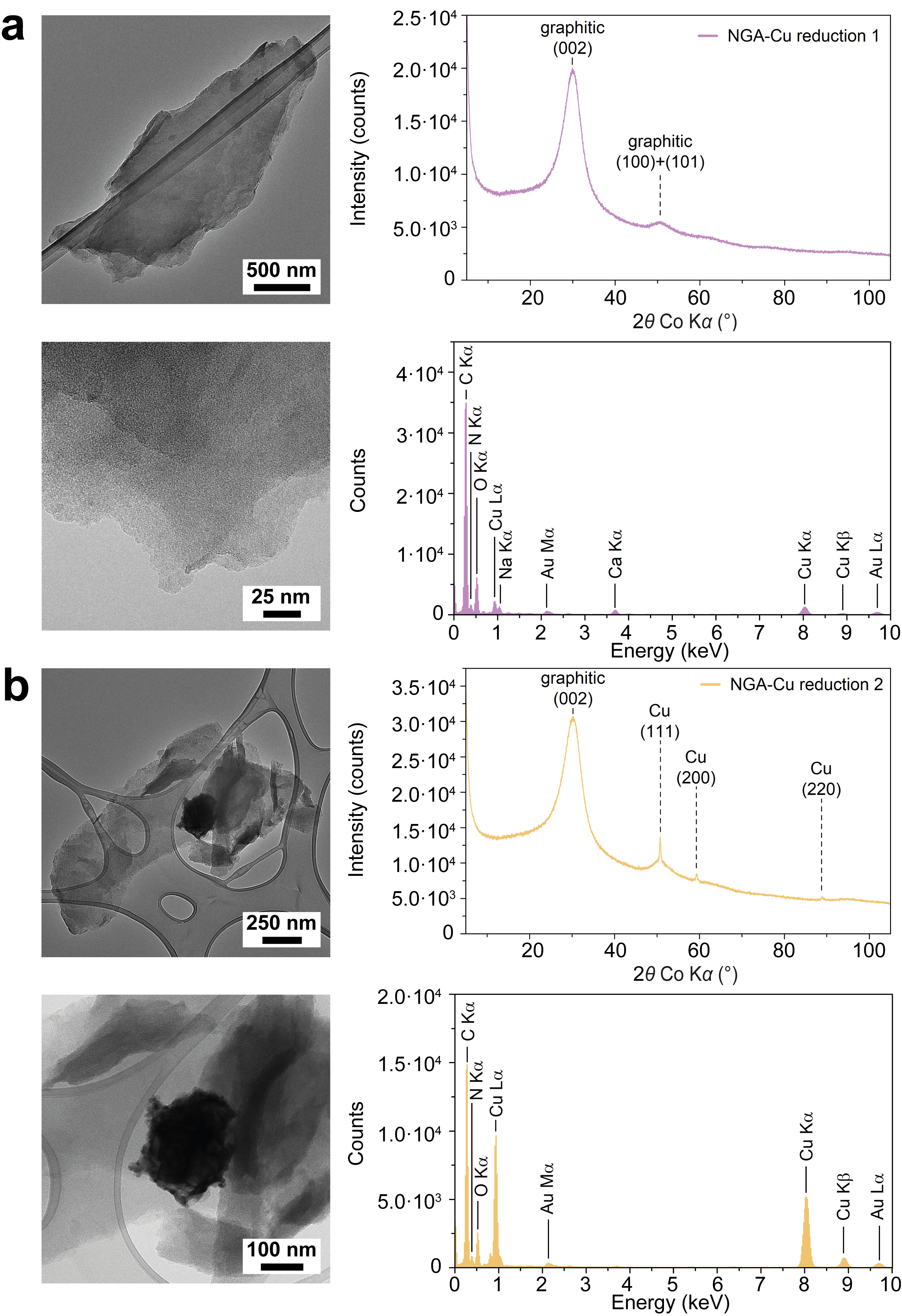


**Figure S5.** a) TEM images, XRD spectra and EDS spectra of NGA-Cu material after reduction process 1 (addition of NaBH_4_ 40 mmol·L^−1^ at room temperature, 30 min of stirring). b) TEM images, XRD spectra and EDS spectra of NGA-Cu material after reduction process 2 (addition of hydrazine 60% at 100 °C, 60 min of stirring^[1]^).


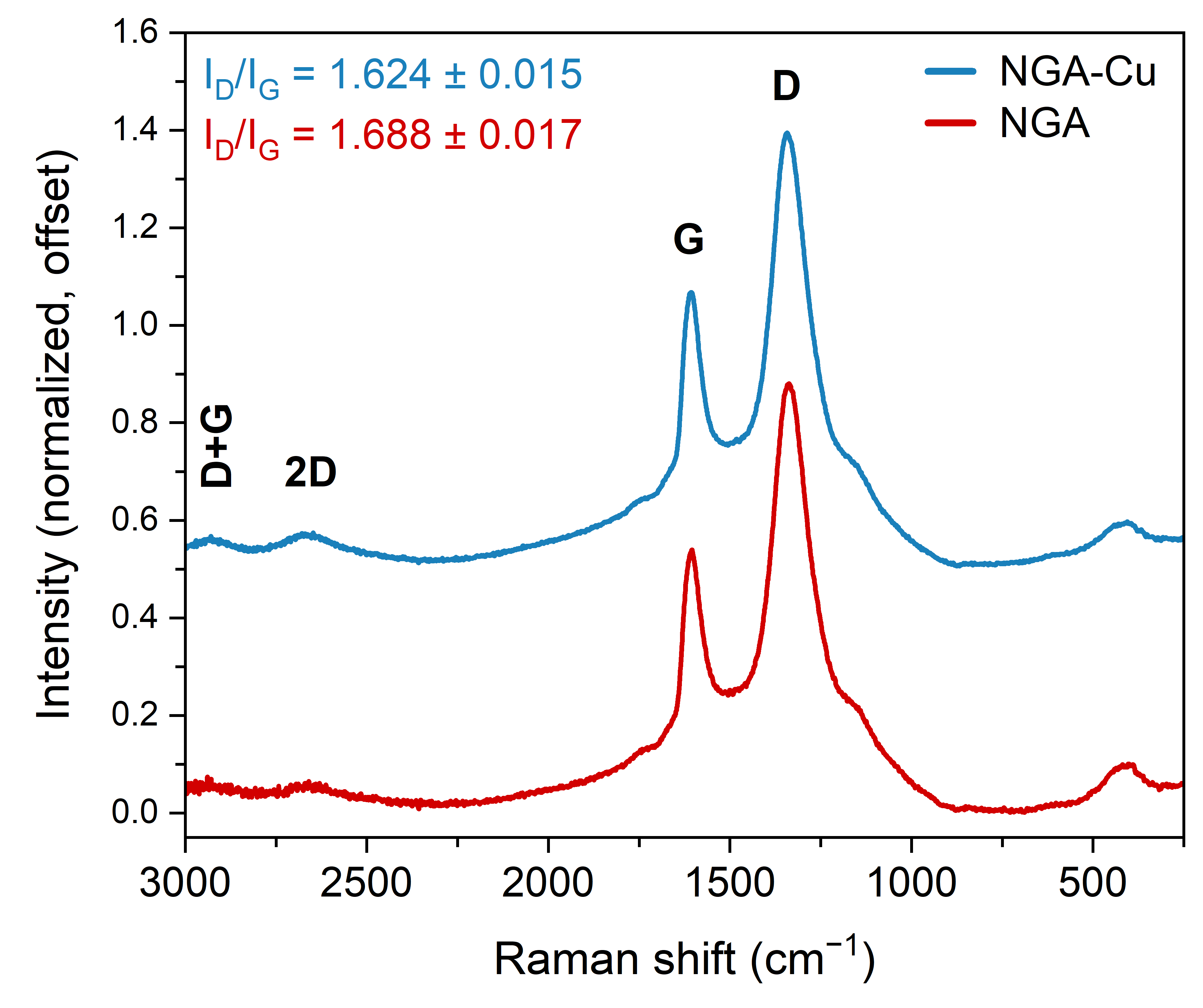


**Figure S6.** Raman spectra of NGA-ink (red line) and NGA-Cu-ink (blue line) with assigned D, G, 2D, and D+G bands and calculated I_D_/I_G_ ratios.


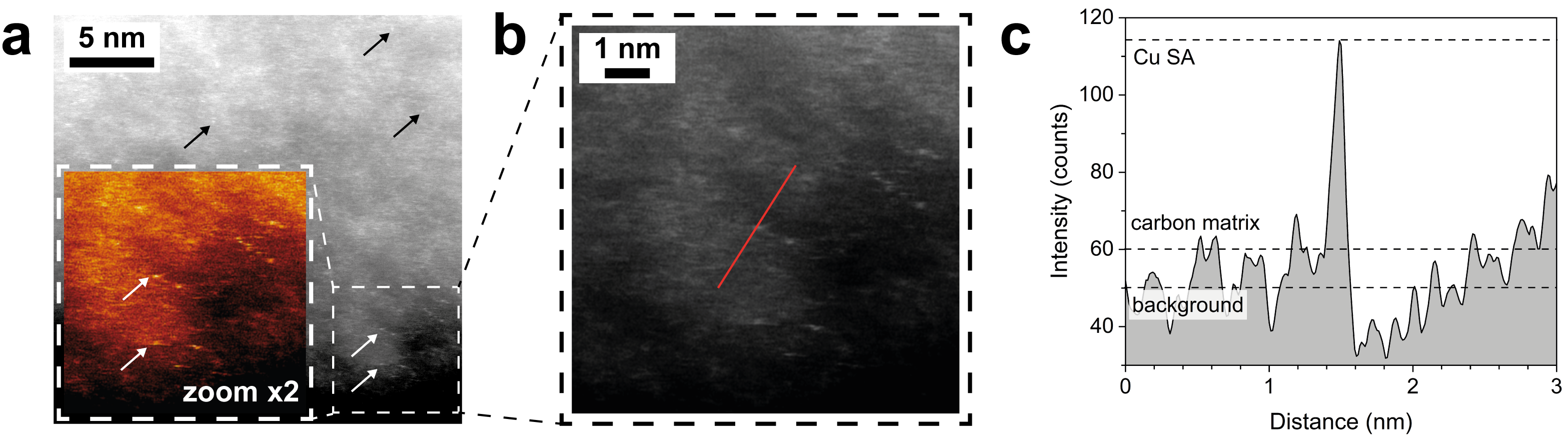


**Figure S7.** HAADF-STEM contrast analysis of isolated bright features in NGA-Cu-ink. a) HAADF-STEM image showing isolated bright spots assigned to individual Cu atoms dispersed on the NGA matrix. b) Magnified region used for contrast analysis, with the red line indicating the evaluated intensity profile. c) Corresponding intensity profile showing the background and carbon matrix intensity, with the pronounced local maximum assigned to a Cu single atom.

The background intensity level in the analyzed image is approximately 50 counts, while the local maxima assigned to isolated bright features reach approximately 114 counts. The surrounding carbon matrix shows typical local intensities of approximately 60 counts. After background subtraction, this corresponds to an apparent Cu/C contrast ratio of approximately 6.4, estimated as:

$$\frac{I_{Cu}}{I_{C}}=\frac{114-50}{60-50}=6.4$$

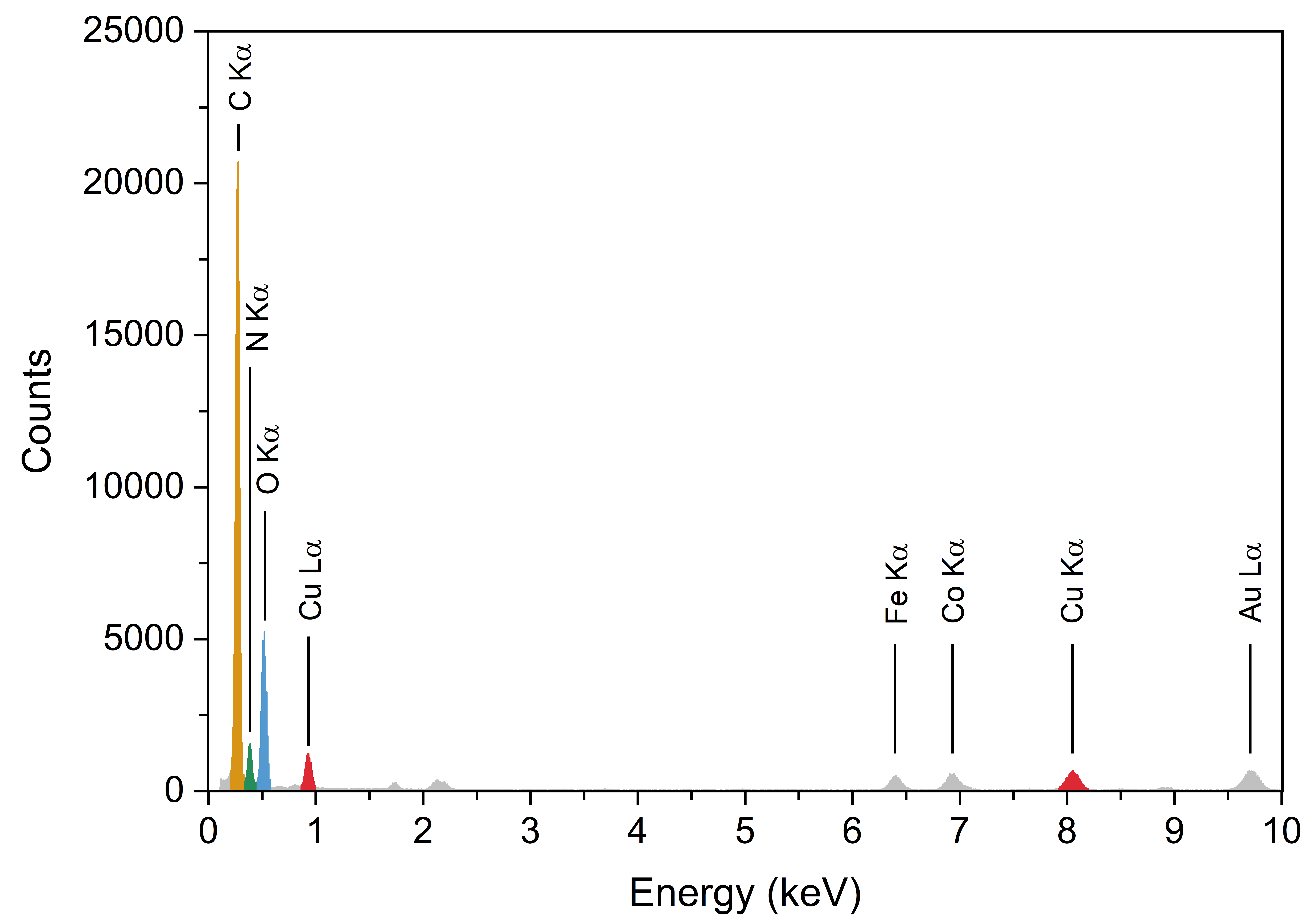


**Figure S8.** EDS spectrum of NGA-Cu-ink. Yellow = C K*α* emission, green = N K*α* emission, blue = O K*α* emission, and red = Cu K/L*α* emission. Background (grey) signals of Fe, Co, and Au are present because of the materials of the holder, grid, and pole piece.


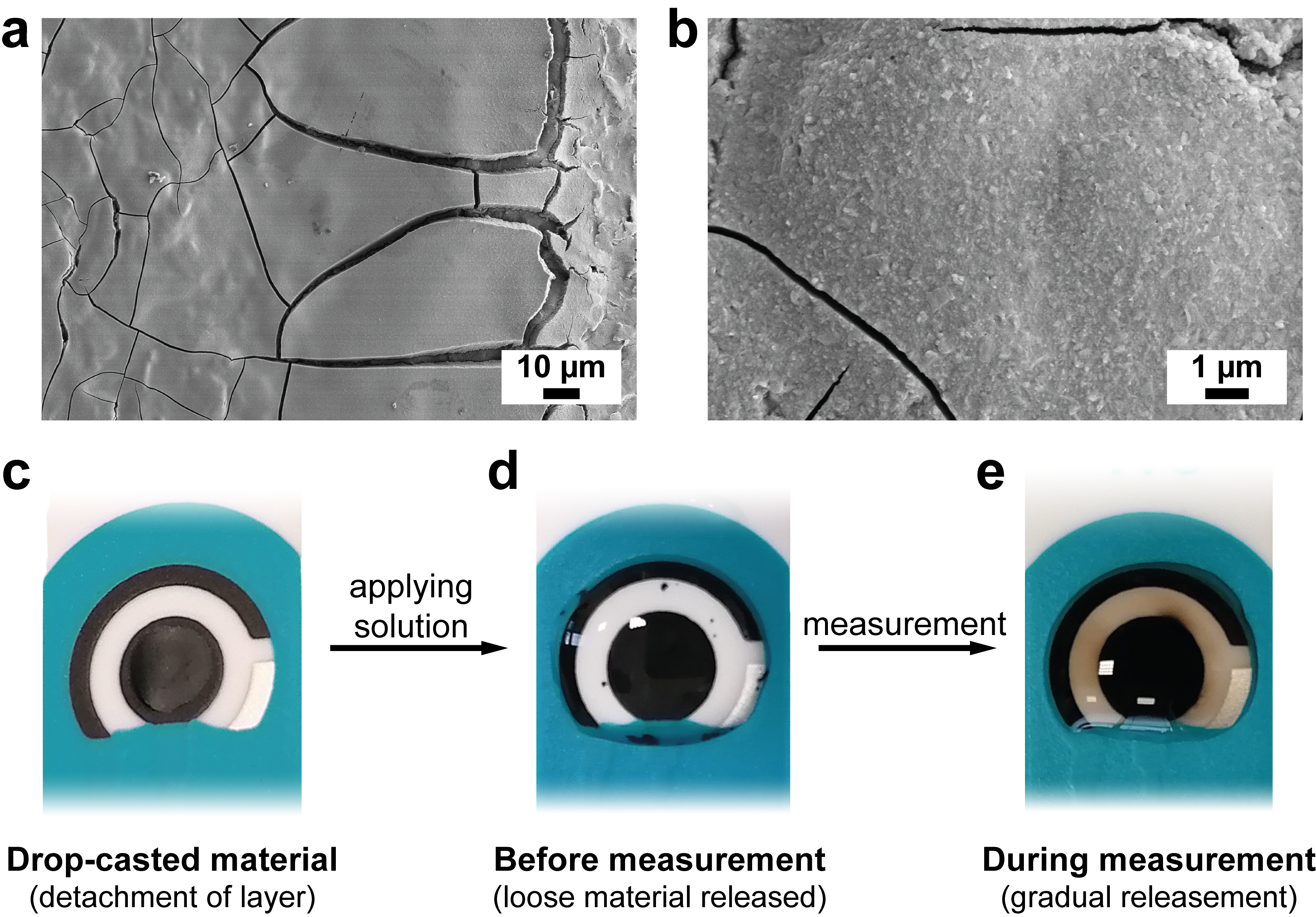


**Figure S9.** a–b) SEM images of drop-casted layer of NGA-Cu-ink on SPCE with visible cracks and particle aggregation. Photos showing adverse effects of drop-casting of graphene materials that affect electrochemical measurements: c) detachment of dried drop-casted layer from the electrode surface, d) immediate release of loose aggregates into measured solution, and e) gradual release of particles into measured solution during the measurement.


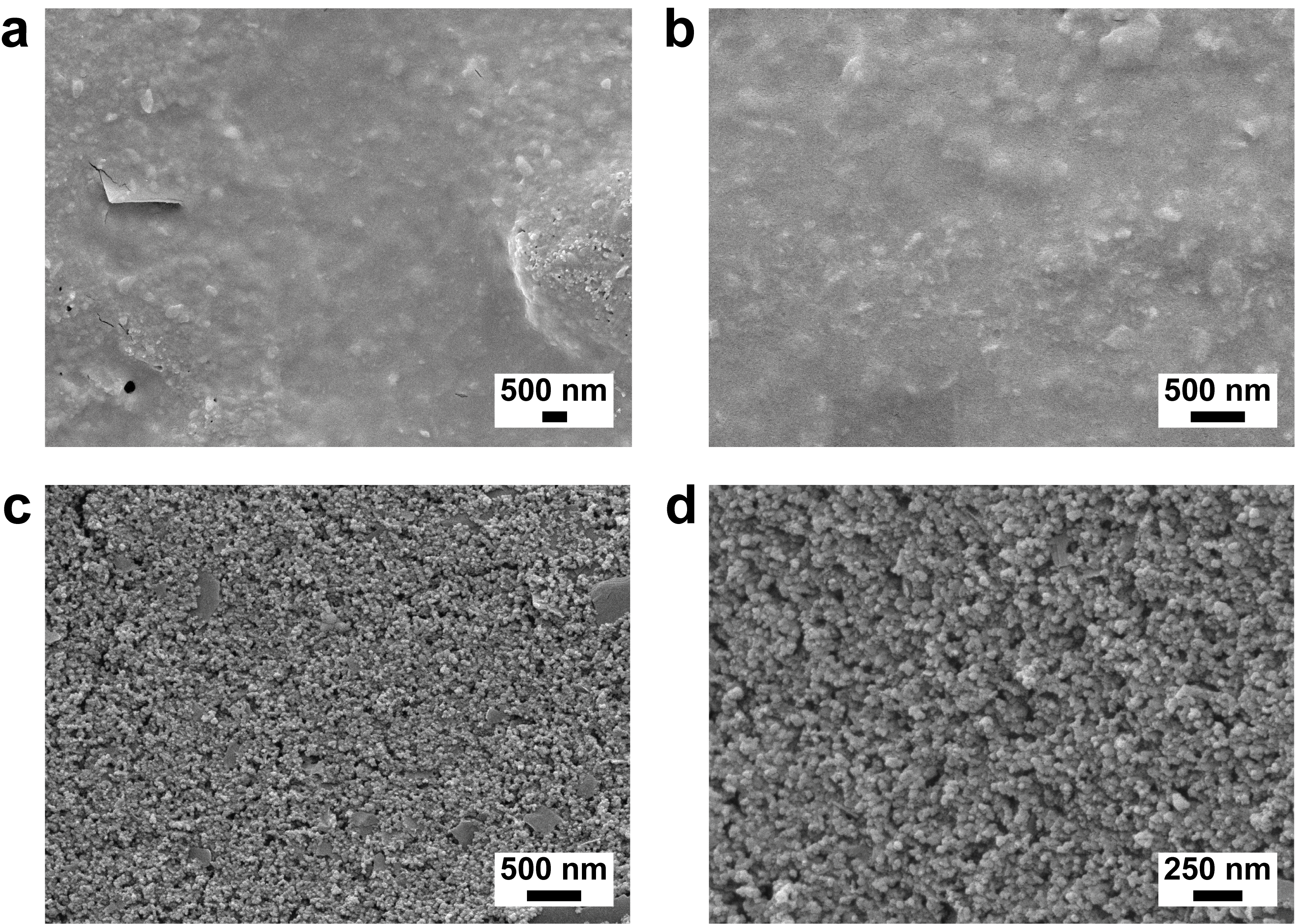


**Figure S10.** a–b) SEM images of ten inkjet-printed layers of NGA-Cu-ink on SPCE. c–d) SEM images of bare SPCE surface.

**Table S1.** Relevant chemical properties of single atoms selected to functionalize NGA-ink.

|  | **_25_Mn** | **_26_Fe** | **_29_Cu** | **_58_Ce** |
| --- | --- | --- | --- | --- |
| Common positive oxidation states | +2, +3, +4, +7 | +2, +3 | +1, +2 | +3, +4 |
| Atomic radius [pm] | 140 | 140 | 135 | 185 |
| Valence shell configuration | 3d^5^ 4s^2^ | 3d^6^ 4s^2^ | 3d^10^ 4s^1^ | 4f^1^ 5d^1^ 6s^2^ |
| Redox potential  (vs SHE) | +1.510  Mn^3+^ + e^−^ ⇌ Mn^2+^ | +0.771  Fe^3+^ + e^−^ ⇌ Fe^2+^ | +0.153  Cu^2+^ + e^−^ ⇌ Cu^+^ | +1.610  Ce^4+^ + e^−^ ⇌ Ce^3+^ |


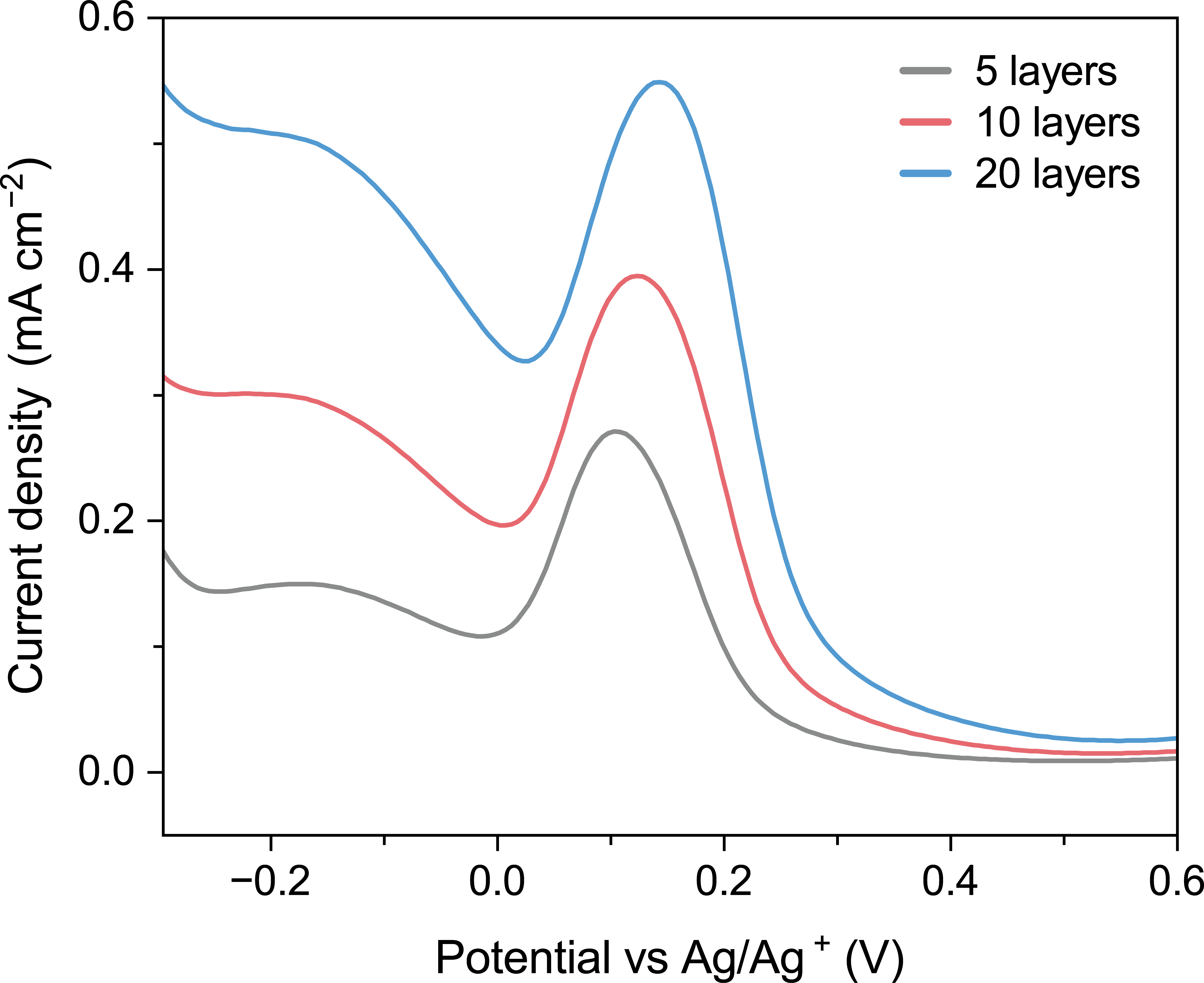


**Figure S11.** Differential pulse voltammetry (DPV) responses of SPCEs modified with 5, 10 and 20 inkjet-printed layers of NGA-ink. Measurements were performed with dopamine solution (1 mmol·L^−1^ in PBS buffer). Full electrochemical operating parameters are provided in Table S4.

**Table S2.** R_CT_ and R_S_ values extracted by fitting of electrochemical impedance spectroscopy (EIS) measurements (5 mmol·L^−1^ K_3_[Fe(CN)_6_] in 0.1 mol·L^−1^ KCl) of SPCEs modified with NGA-ink variants. Values are reported as mean ± SD from three independently prepared electrodes.

| **Material** | **R_CT_ [Ω]** | **R_S_ [Ω]** |
| --- | --- | --- |
| SPCE bare | 328 ± 41 | 112 ± 3 |
| SPCE NGA-ink | 457 ± 7 | 110 ± 1 |
| SPCE NGA-Cu-ink | 985 ± 75 | 104 ± 2 |
| SPCE NGA-Mn-ink | 773 ± 54 | 109 ± 3 |
| SPCE NGA-Fe-ink | 748 ± 32 | 106 ± 1 |
| SPCE NGA-Ce-ink | 548 ± 67 | 115 ± 1 |


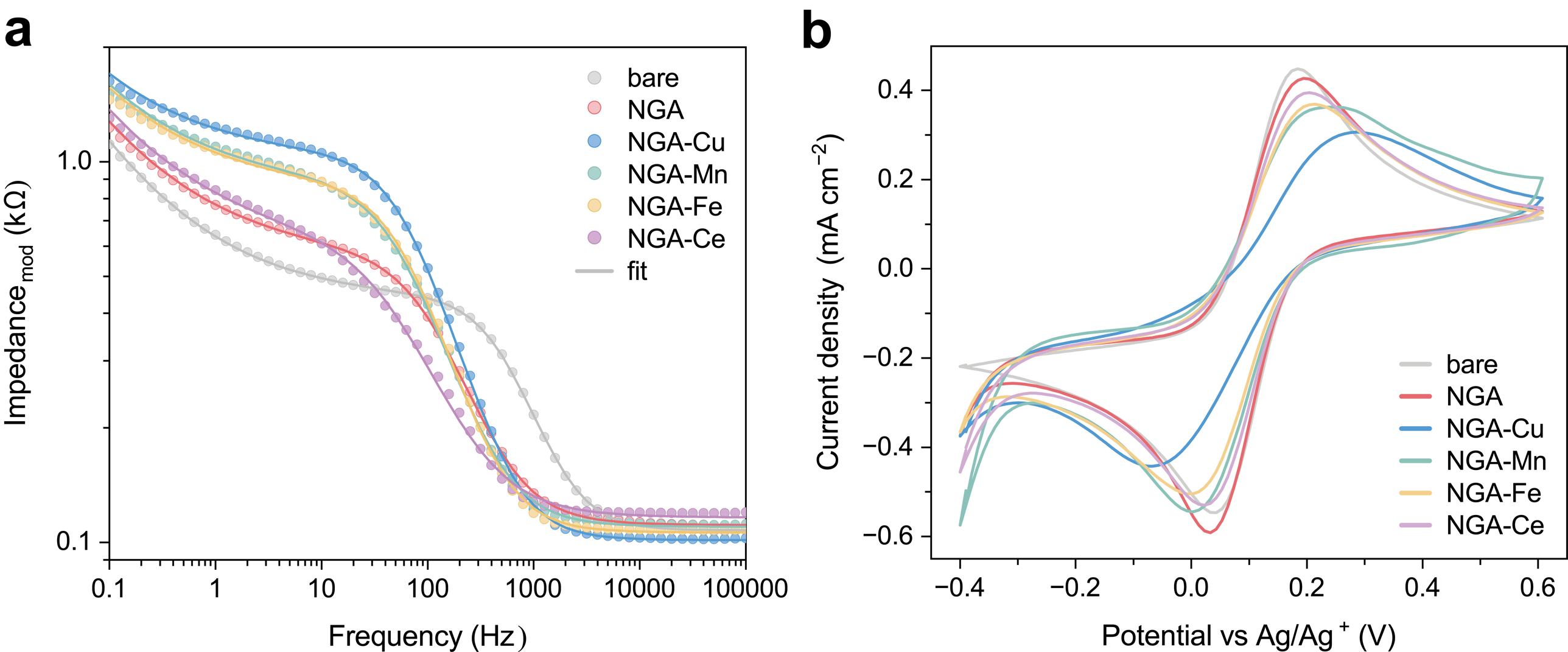


**Figure S12.** a) Bode plots showing the frequency-dependent impedance responses (5 mmol·L^−1^ K_3_[Fe(CN)_6_] in 0.1 mol·L^−1^ KCl) of the bare SPCE and SPCEs modified with inkjet-printed NGA-ink variants. b) Complementary voltammograms (scan rate 0.05 V·s^−1^) showing responses (5 mmol·L^−1^ K_3_[Fe(CN)_6_] in 0.1 mol·L^−1^ KCl) of the bare SPCE and SPCEs modified with inkjet-printed NGA-ink variants. Full electrochemical operating parameters are provided in Table S4.

**Table S3.** Calculated k^0^ values for bare SPCE and SPCEs modified with inkjet-printed NGA-ink variants. k^0^ values were calculated from the mean R_CT_ values obtained from three independently prepared electrodes.

| **Material** | **k^0^ [cm·s^−1^]** |
| --- | --- |
| SPCE bare | 1.29·10^−3^ |
| SPCE NGA-ink | 9.27·10^−4^ |
| SPCE NGA-Cu-ink | 4.30·10^−4^ |
| SPCE NGA-Mn-ink | 5.48·10^−4^ |
| SPCE NGA-Fe-ink | 5.66·10^−4^ |
| SPCE NGA-Ce-ink | 7.74·10^−4^ |





**Figure S13.** Elimination voltammetry with linear scan (EVLS) voltammograms depicting diffusion (I_d_), kinetic (I_k_) and capacitive (I_c_) current components of bare SPCE (a) and SPCEs modified with b) NGA-ink, c) NGA-Cu-ink, d) NGA-Mn-ink, e) NGA-Fe-ink, and f) NGA-Ce-ink.

**Table S4.** Electrochemical operating parameters used for EIS, cyclic voltammetry (CV), and DPV measurements. The parameters are grouped according to the electrochemical technique and the corresponding figures. Potentials are reported versus the respective reference electrode used in each electrode setup.

| **EIS parameters** | | | | |
| --- | --- | --- | --- | --- |
| **Figures** | **DC potential** | **Frequency range** | **AC amplitude** | **Other parameters** |
| Fig. 4a, b;  Fig. S12a | approximated as E_1/2_=(E_pa_+E_pc_)/2 | 100 kHz–0.1 Hz | 0.01 V_RMS_ | sinusoidal perturbation, 10 frequencies per decade |
| **CV parameters** | | | | |
| **Figures** | **Scan rate** | **Step potential** | **Number of scans** |  |
| Fig. S12b, S16b, S17b, S18, S20 | 0.05 V·s^−1^ | 0.01 V | 3 |  |
| **DPV parameters** | | | | |
| **Figures** | **Modulation amplitude** | **Step potential** | **Modulation time** | **Other parameters** |
| Fig. 5a–d;  Fig. S11, S14, S15, S16a, c, S17a | 0.025 V | 0.005 V | 0.05 s | interval time 0.5 s, effective scan rate 0.01 V·s^−1^ |
| Fig. 6c–e;  Fig. S21, S22 | 0.050 V | 0.005 V | 0.05 s | interval time 0.5 s, effective scan rate 0.01 V·s^−1^ |


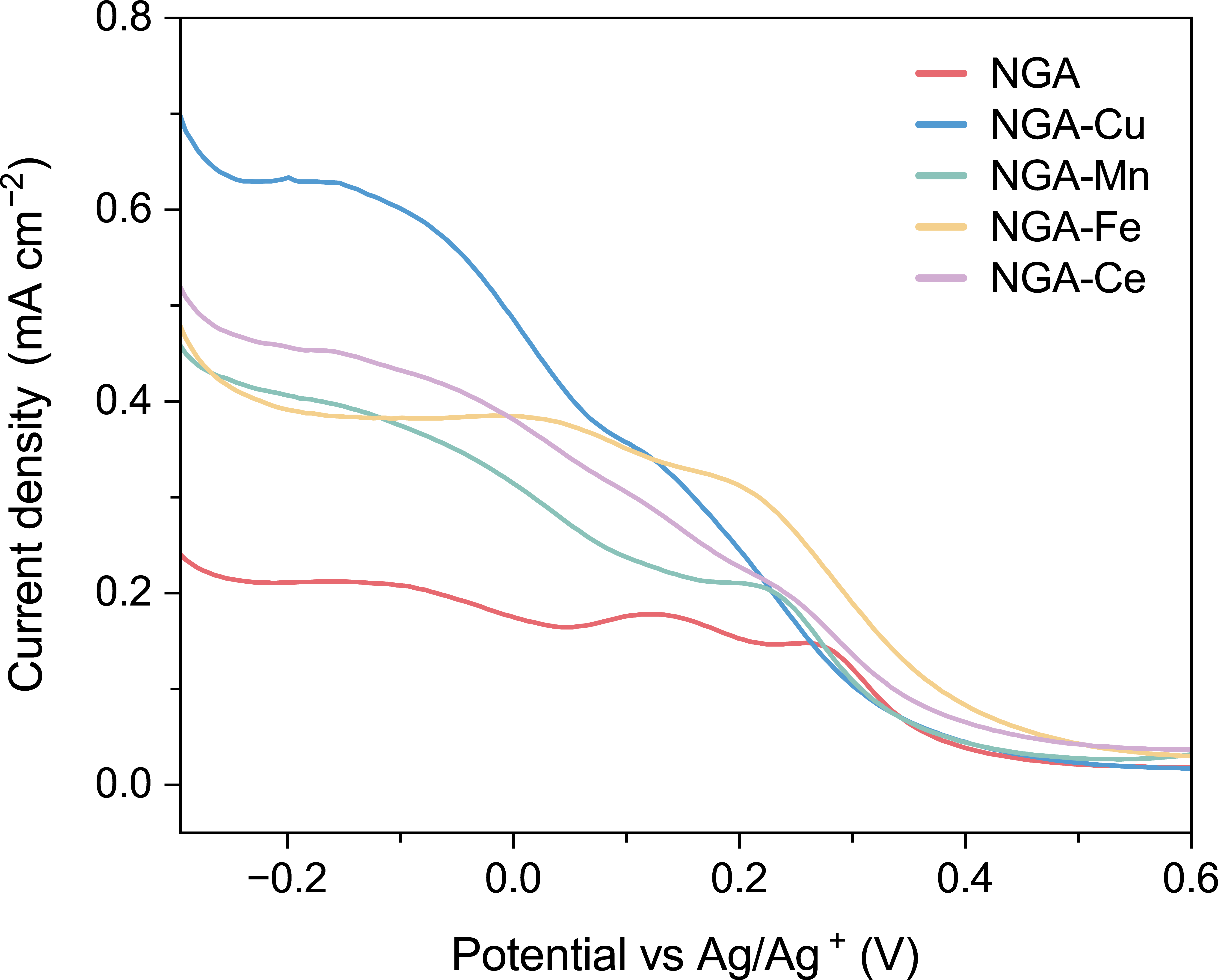


**Figure S14.** DPV responses of SPCEs modified with drop-casted NGA-ink variants in the presence of dopamine (1 mmol·L^−1^ in PBS buffer). Full electrochemical operating parameters are provided in Table S4.





**Figure S15.** DPV responses in the presence of dopamine (1 mmol·L^−1^ in PBS buffer; solid line) and PBS buffer (dotted line) of a) bare SPCE, and SPCEs modified with inkjet-printed b) NGA-ink, c) NGA-Cu-ink, d) NGA-Mn-ink; e) NGA-Fe-ink, and f) NGA-Ce-ink. Full electrochemical operating parameters are provided in Table S4. DPV responses were baseline-corrected by polynomial fitting.

**Table S5.** Calculated apparent electrochemical surface area (ECSA_app_) values for bare SPCE and SPCEs modified with inkjet-printed NGA-ink variants.

| **Material** | **ECSA_app_ [cm^2^]** |
| --- | --- |
| SPCE bare | 0.0890 |
| SPCE NGA-ink | 0.0848 |
| SPCE NGA-Cu-ink | 0.0608 |
| SPCE NGA-Mn-ink | 0.0720 |
| SPCE NGA-Fe-ink | 0.0732 |
| SPCE NGA-Ce-ink | 0.0784 |

Apparent electroactive surface areas were determined from the scan-rate-dependent cyclic voltammetric response of 5 mmol·L^−1^ K_3_[Fe(CN)_6_] in 0.1 mol·L^−1^ KCl. Because several modified electrodes exhibited pseudo-reversible rather than fully reversible ferricyanide behavior, as evidenced by broadened peak separation and altered peak shapes in **Figure S12b,** the classical Randles–Ševčík equation for reversible systems was not used. Instead, the apparent ECSA values were calculated using the modified Randles–Ševčík relation for pseudo-reversible diffusion-controlled systems:

$$I_{p}=2.99\times{10}^{5}n(\alpha n_{\alpha})^{1/2}ACD^{1/2}\upsilon^{1/2}$$

where *I_p_* is the anodic peak current, *n* is the number of transferred electrons, *α* is the charge-transfer coefficient, *n_α_* is the number of electrons involved in the rate-determining step, *A* is the apparent electroactive surface area, *C* is the concentration of the redox probe, *D* is its diffusion coefficient, and *υ* is the scan rate. The values reported in **Table S5** should therefore be regarded as apparent comparative ECSA values, ECSA_app_, rather than absolute electroactive areas.

**Table S6.** Average peak current density values of SPCEs modified with NGA-ink variants and relative enhancement of the signal compared to the response of pristine NGA-ink. Data were extracted from DPV measurements of dopamine (1 mmol·L^−1^ in PBS buffer, pH = 7.4) using three independently prepared electrodes for each material and are reported as mean ± SD.

| **Material** | **Average peak current density [mA·cm^−2^]** | **Relative signal change compared to NGA-ink [%]** |
| --- | --- | --- |
| SPCE NGA-ink | 0.194 ± 0.002 | 100.00 |
| SPCE NGA-Cu-ink | 0.236 ± 0.002 | 121.54 |
| SPCE NGA-Mn-ink | 0.176 ± 0.003 | 90.64 |
| SPCE NGA-Fe-ink | 0.144 ± 0.002 | 74.21 |
| SPCE NGA-Ce-ink | 0.122 ± 0.002 | 63.07 |





**Figure S16.** Effect of Cu loading in NGA-Cu-ink on dopamine oxidation at inkjet-modified SPCEs. a) DPV and b) CV responses of SPCEs modified with inkjet-printed NGA-Cu-ink containing different Cu contents compared with pristine NGA-ink, measured in PBS and in dopamine solution (1 mmol·L^−1^ in PBS buffer). Full electrochemical operating parameters are provided in Table S4. DPV responses were baseline-corrected by polynomial fitting. c) Corresponding dopamine peak current densities extracted from DPV measurements. Data are presented as mean ± SD from three independently prepared electrodes.


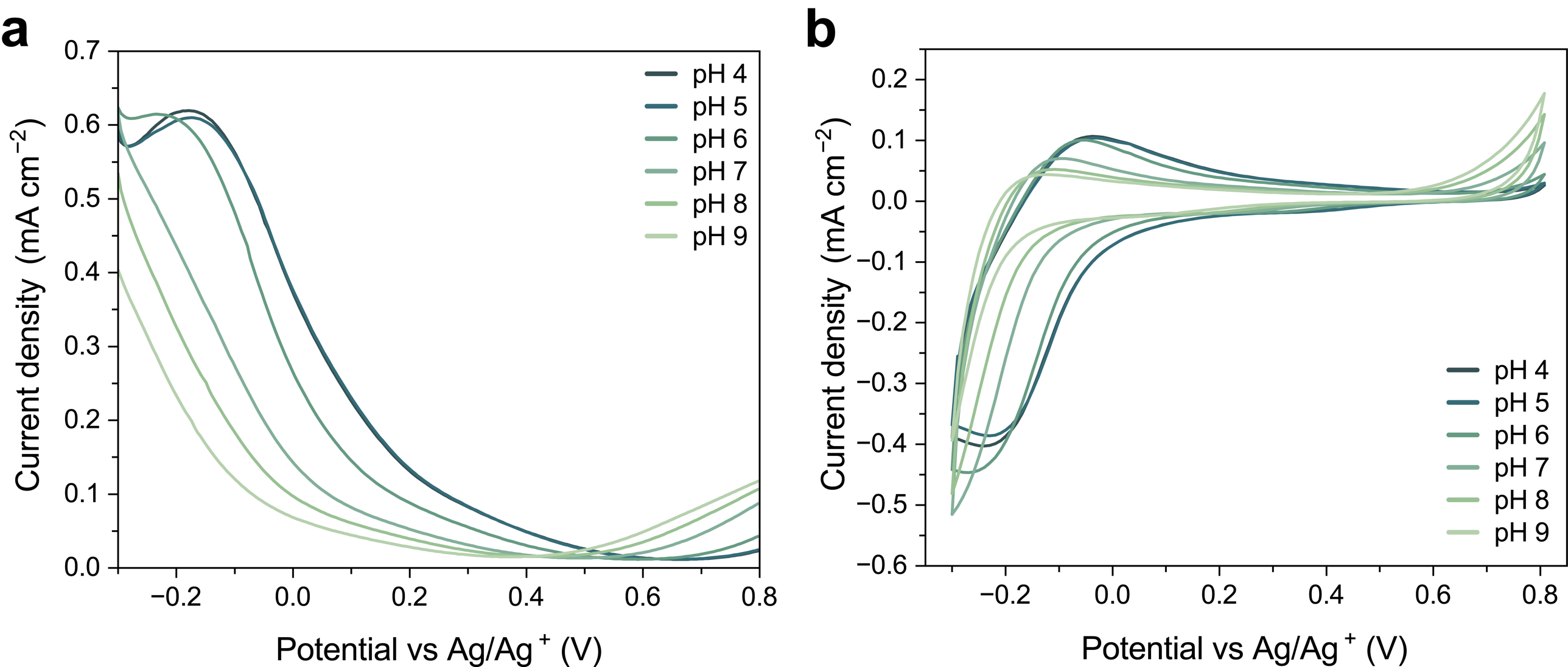


**Figure S17.** pH-dependent electrochemical response of SPCEs modified with inkjet-printed NGA-Cu-ink in Britton–Robinson buffer. a) DPV and b) CV responses recorded over the pH range 4–9. Full electrochemical operating parameters are provided in Table S4.

**Table S7.** Comparison of performance metrics of previously published works on dopamine sensing that include single-atom systems based on N-doped carbon.

| **Substrate** | **Single atom** | **LoD [μmol·L^−1^]** | **Sensitivity [μA·μM^−1^·cm^−2^]** | **Reference** |
| --- | --- | --- | --- | --- |
| N-doped carbon | Ni | 0.027 | – | Qin et al.^[2]^ |
| N-doped carbon | Co | 0.04 | 0.98 | Shu et al.^[3]^ |
| N-doped carbon | Co | 0.049 | – | Liu et al.^[4]^ |
| N-doped carbon | Fe, Pt | 0.27 | – | Zhang et al.^[5]^ |
| N-doped carbon | Co | 0.32 | – | Liu et al.^[6]^ |
| hollow N-doped carbon | Fe | 0.32 | – | Wang et al.^[7]^ |
| N-doped carbon | Co | 1.36 | – | Jin et al.^[8]^ |
| N,S-co-doped carbon | Fe | 3.76 | 0.02 | Gao et al.^[9]^ |
| **N-doped graphene acid** | **Cu** | **10.6** | **0.70** | **this work** |


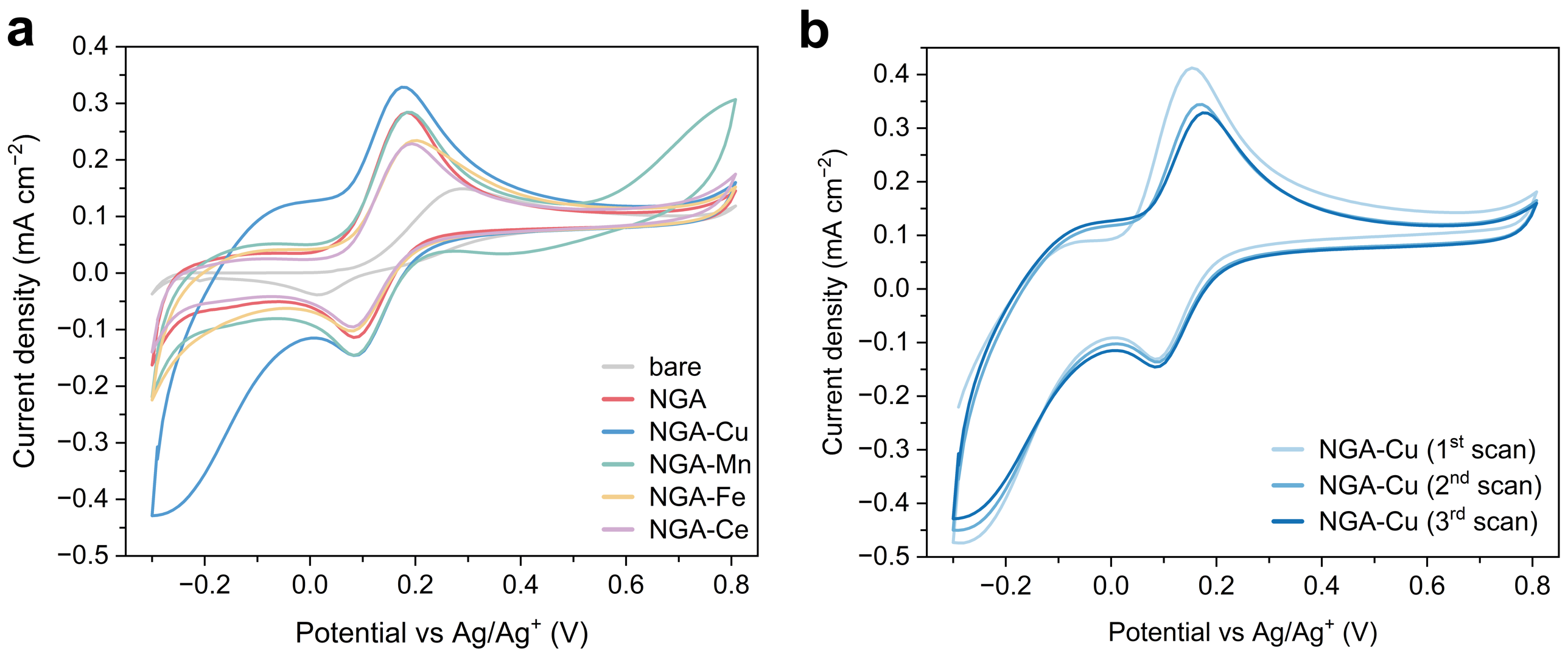


**Figure S18.** a) CV responses of bare SPCE and SPCEs modified with inkjet-printed NGA-ink variants in the presence of dopamine (1 mmol·L^−1^ in PBS buffer). Full electrochemical operating parameters are provided in Table S4. b) Consecutive CV scans of a NGA-Cu-ink-modified SPCE in the presence of dopamine (1 mmol·L^−1^ in PBS buffer), showing a stable dopamine redox couple without the emergence of additional peaks associated with dopaminochrome or polydopamine formation.


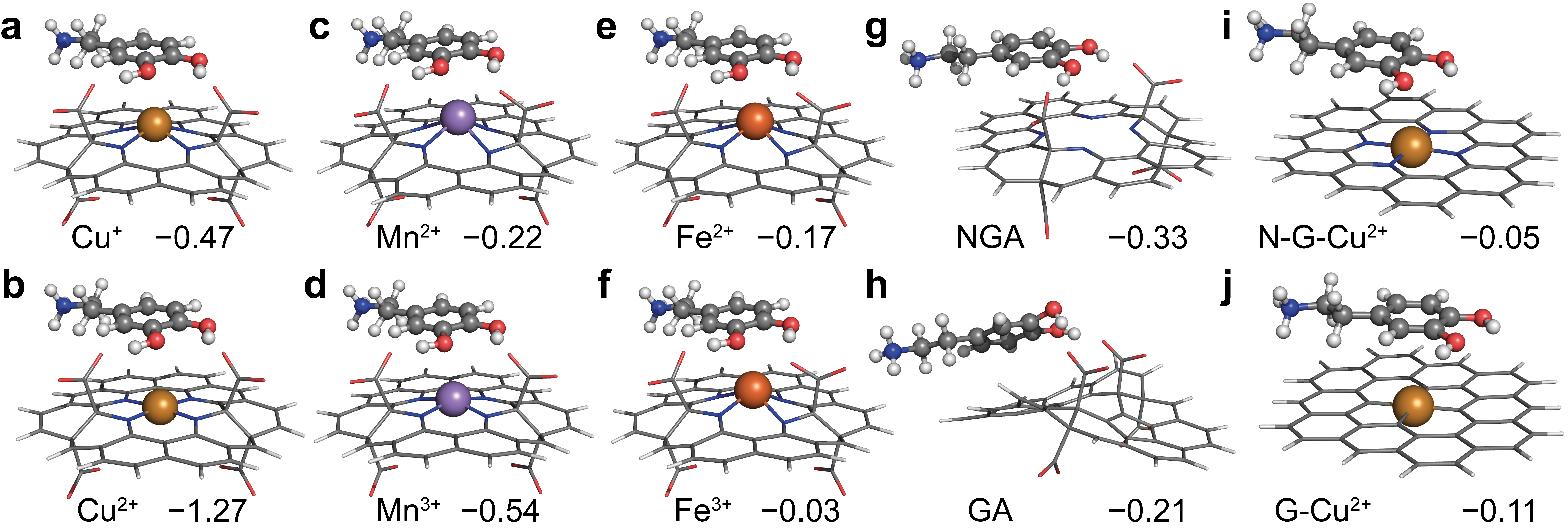


**Figure S19.** Optimized structural models with dopamine adsorbed at a) NGA-Cu^+^, b) NGA-Cu^2+^, c) NGA-Mn^2+^, d) NGA-Mn^3+^, e) NGA-Fe^2+^, f) NGA-Fe^3+^, (g) NGA, (h) graphene acid (GA), (i) N-doped graphene with anchored Cu (N-G-Cu^2+^), and (j) graphene with anchored Cu (G-Cu^2+^). Adsorption free energies of dopamine at NGA-SA are given in eV. Color scheme: Cu-brown, Mn-purple, Fe-ochre, C-gray, N-blue, O-red, H-white.

**Table S8.** Calculated production cost of one individual fully inkjet-printed NGA-Cu electrode, including all ink and substrate consumption.

| **Material** | **Amount** | | **Price/unit** | | **Price** | |
| --- | --- | --- | --- | --- | --- | --- |
| Ag nanoparticle ink | 241 881 | drops | 3.25 | $/mL | 0.0019 | $ |
|  | 0.5805 | μL |  |  |  |  |
| Au nanoparticle ink | 184 335 | drops | 55.56 | $/mL | 0.0246 | $ |
|  | 0.4424 | μL |  |  |  |  |
| NGA-Cu-ink | 272 763 | drops | 4 | $/mL | 0.0026 | $ |
|  | 0.6546 | μL |  |  |  |  |
| Dielectric ink | 953 373 | drops | 4.65 | $/mL | 0.0106 | $ |
|  | 2.2881 | μL |  |  |  |  |
| Photopaper (substrate) | 1.60 | cm^2^ | 0.0013 | $/cm^2^ | 0.0021 | $ |
| **Electrode price** |  |  |  |  | 0.0418 | $ |


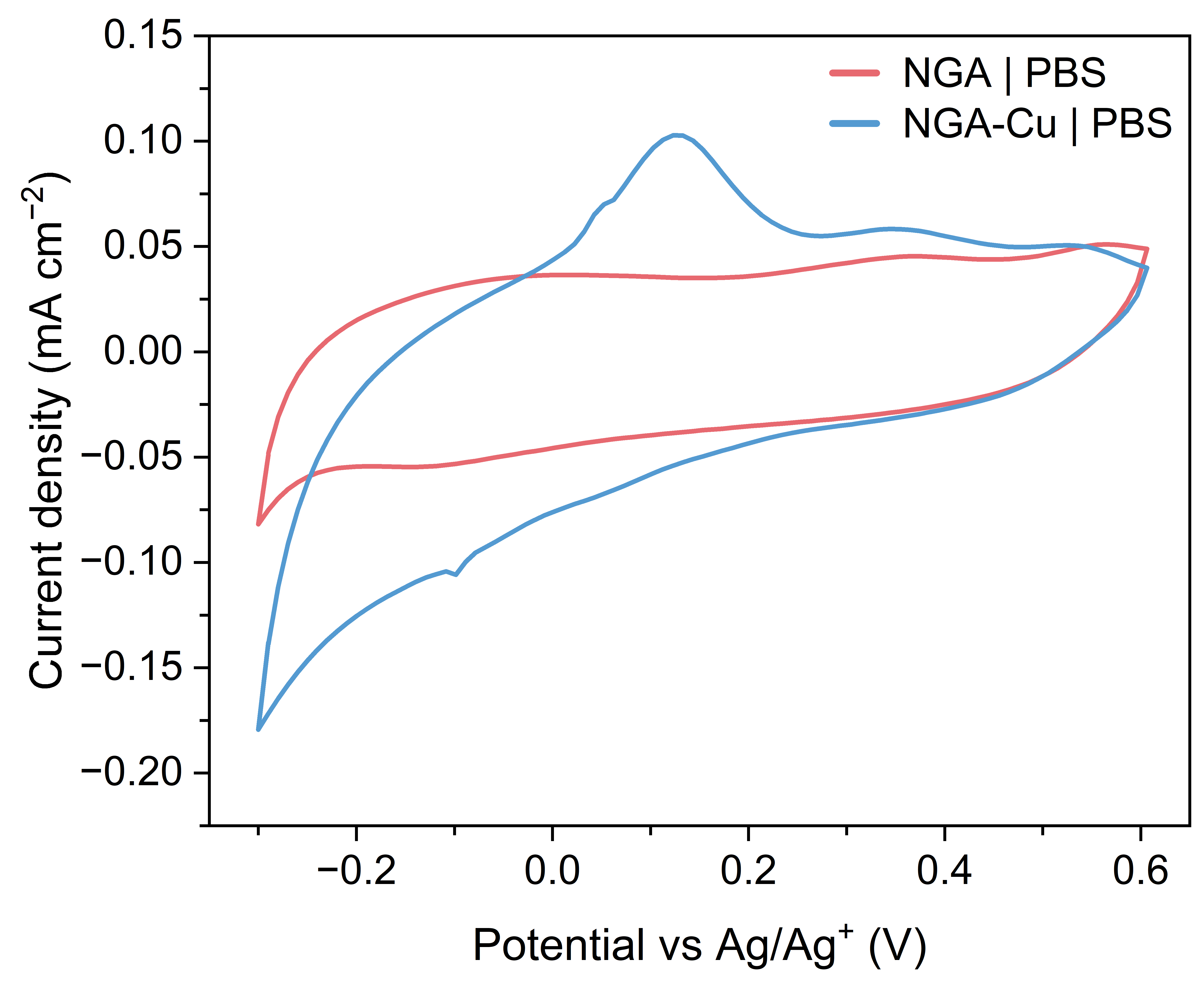


**Figure S20.** CV responses of fully inkjet-printed electrodes modified with inkjet-printed NGA-ink and NGA-Cu-ink in the presence of PBS buffer. Full electrochemical operating parameters are provided in Table S4.





**Figure S21.** DPV responses of fully inkjet-printed electrodes modified with NGA-ink (grey lines) and NGA-Cu-ink (lines of color) in the presence of PBS buffer (blank sample) and a) uric acid (1 mmol·L^−1^ in PBS buffer), b) serotonin (1 mmol·L^−1^ in PBS buffer), c) melatonin (1 mmol·L^−1^ in PBS buffer), and d) ascorbic acid (1 mmol·L^−1^ in PBS buffer). e) DPV responses of fully inkjet-printed electrodes modified with NGA-Cu-ink in the presence of PBS buffer, dopamine, and selected interferents. Full electrochemical operating parameters are provided in Table S4. DPV responses were baseline-corrected by polynomial fitting.


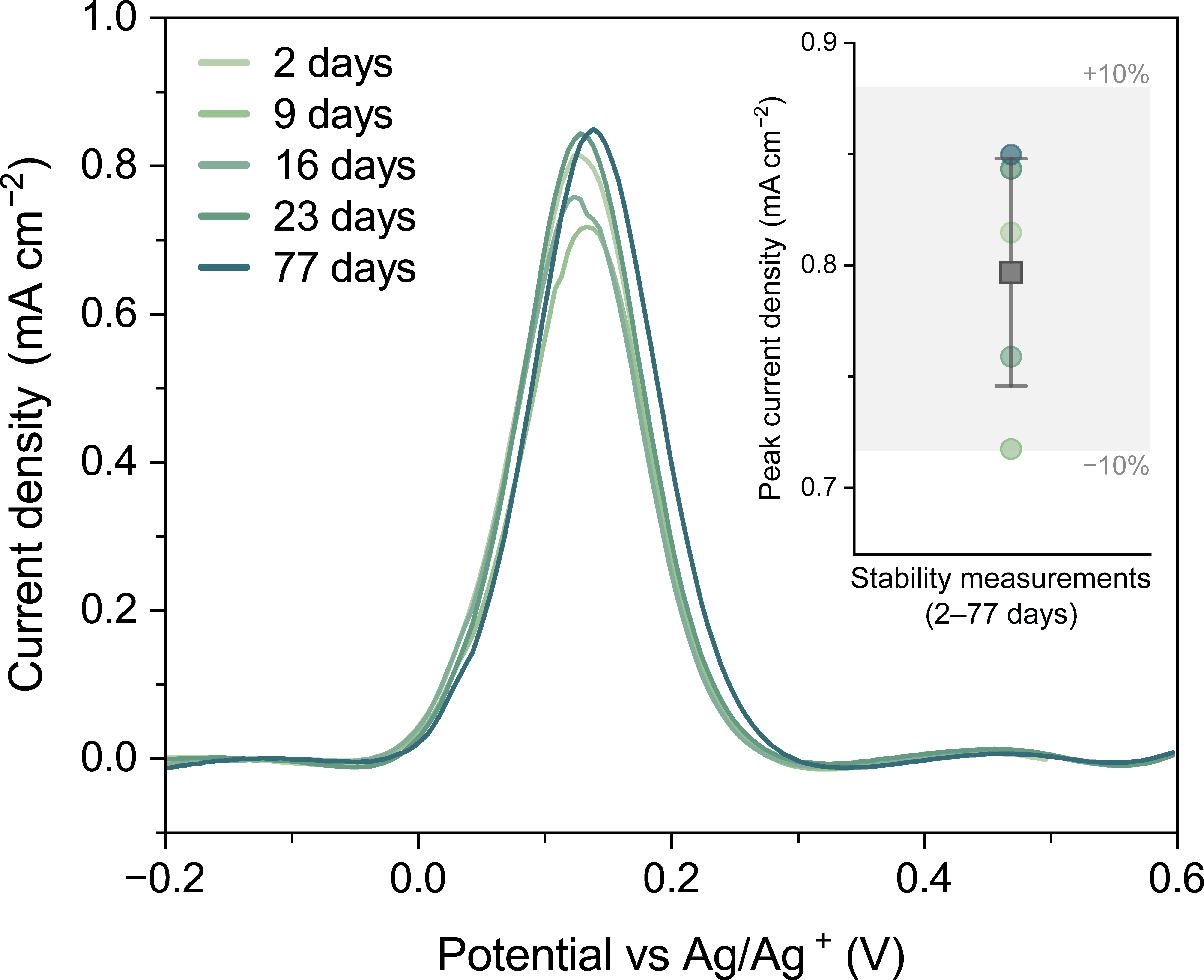


**Figure S22.** DPV responses of fully inkjet-printed electrodes modified with inkjet-printed NGA-Cu-ink in the presence of dopamine (1 mmol·L^−1^ in PBS buffer), reflecting their stability over a period of 11 weeks. Inset shows that all peak current densities, extracted from the measurements, lie within ±10% range from the average value. Full electrochemical operating parameters are provided in Table S4. DPV responses were baseline-corrected by polynomial fitting.

**References**

1. K. Kim, K. N. Chaudhari, S. Kim, Y. Kim, and K. S. Shin, “Facile Single-Step Synthesis of Cu-rGO Nanocomposite through Simultaneous Reduction Process and Its Peroxidase Mimic Activity,” *Journal of Industrial and Engineering Chemistry* *95* (2021): 388–396, https://doi.org/10.1016/j.jiec.2021.01.013.

2. Z. Qin, B. Tang, G. Zhang, et al., “Single-Atom Ni-N4 for Enhanced Electrochemical Sensing,” *Nano Research* *17*, no. 8 (2024): 7658–7664, https://doi.org/10.1007/s12274-024-6771-6.

3. Y. Shu, Z. Li, Y. Yang, et al., “Isolated Cobalt Atoms on N-Doped Carbon as Nanozymes for Hydrogen Peroxide and Dopamine Detection,” *ACS Applied Nano Materials* *4*, no. 8 (2021): 7954–7962, https://doi.org/10.1021/acsanm.1c01278.

4. Y. Liu, Y. Zhang, C. Wang, et al., “Co Single-Atom Nanozymes for the Simultaneous Electrochemical Detection of Uric Acid and Dopamine in Biofluids,” *ACS Applied Nano Materials* *7*, no. 6 (2024): 6273–6283, https://doi.org/10.1021/acsanm.3c06213.

5. Y. Zhang, X. Liu, J. Zhao, et al., “Adjacent Fe-Pt Atomic Site Synergistically Boosting Wearable Biosensor Performance in Sweat Analysis,” *Chemical Engineering Journal* *499* (2024): 155991, https://doi.org/10.1016/j.cej.2024.155991.

6. Y. Liu, P. Zhao, Y. Liang, et al., “Single-Atom Nanozymes Co–N–C as an Electrochemical Sensor for Detection of Bioactive Molecules,” *Talanta* *254* (2023): 124171, https://doi.org/10.1016/j.talanta.2022.124171.

7. X. Wang, J. Wang, J. Yang, H. Ding, and Y. Dong, “Isolated Iron Atoms Anchored on MOF-Derived Hollow N-Doped Carbon for the Detection of Dopamine,” *Microchemical Journal* *212* (2025): 113380, https://doi.org/10.1016/j.microc.2025.113380.

8. J. Jin, J.-J. Wei, Z.-Y. Gu, et al., “Dual-Mode Sensor Based on a Single-Atom Cobalt Catalyst for Simultaneous Electrochemical and Colorimetric Detection of Bioactive Small Molecules,” *Analytical Chemistry* *97*, no. 15 (2025): 8617–8624, https://doi.org/10.1021/acs.analchem.5c01216.

9. Y. Gao, L. Yang, Y. Zhang, Y. Li, and L. Zhang, “Nanoarchitectonics of Fe Single-Atom Catalyst Modified with Flexible Wearable Patch for Sweat Biomarker Analysis,” *Microchemical Journal* *204* (2024): 111147, https://doi.org/10.1016/j.microc.2024.111147.
